# Synthetic glycolate metabolism pathways stimulate crop growth and productivity in the field

**DOI:** 10.1126/science.aat9077

**Published:** 2019-01-04

**Authors:** Paul F. South, Amanda P. Cavanagh, Helen W. Liu, Donald R. Ort

**Affiliations:** 1 Global Change and Photosynthesis Research Unit, United States Department of Agriculture–Agricultural Research Service, Urbana, IL 61801, USA; 2 Carl R. Woese Institute for Genomic Biology, University of Illinois, Urbana, IL 61801, USA; 3 Department of Crop Sciences, University of Illinois, Urbana, IL 61801, USA; 4 Department of Plant Biology, University of Illinois, Urbana, IL 61801, USA

## Abstract

Photorespiration is required in C_3_ plants to metabolize toxic glycolate formed when ribulose-1,5-bisphosphate carboxylase-oxygenase oxygenates rather than carboxylates ribulose-1,5-bisphosphate. Depending on growing temperatures, photorespiration can reduce yields by 20 to 50% in C_3_ crops. Inspired by earlier work, we installed into tobacco chloroplasts synthetic glycolate metabolic pathways that are thought to be more efficient than the native pathway. Flux through the synthetic pathways was maximized by inhibiting glycolate export from the chloroplast. The synthetic pathways tested improved photosynthetic quantum yield by 20%. Numerous homozygous transgenic lines increased biomass productivity between 19 and 37% in replicated field trials. These results show that engineering alternative glycolate metabolic pathways into crop chloroplasts while inhibiting glycolate export into the native pathway can drive increases in C_3_ crop yield under agricultural field conditions.

## Introduction

Meeting food demands for the growing global human population requires improving crop productivity, and large gains are possible through enhancing photosynthetic efficiency. Photosynthesis requires the carboxylation of ribulose-1,5-bisphosphate (RuBP) by ribulose-1,5-bisphosphate carboxylase-oxygenase (RuBisCO), but photorespiration occurs in most plants such as soybean, rice, and wheat (known as C_3_ crops) when RuBisCO oxygenates RuBP instead, requiring costly processing of toxic byproducts such as glycolate. Photorespiration can reduce C_3_ crop photosynthetic efficiency by 20 to 50%. Although various strategies exist for lowering the costs of photorespiration, chamber-and greenhouse-grown plants with altered photorespiratory pathways within the chloroplast have shown promising results, including increased photosynthetic rates and plant size.

## Rationale

To determine if alternative photorespiratory pathways could effectively improve C_3_ field crop productivity, we tested the performance of three alternative photorespiratory pathways in field-grown tobacco. One pathway used five genes from the Escherichia coli glycolate oxidation pathway; a second pathway used glycolate oxidase and malate synthase from plants and catalase from E. coli; and the third pathway used plant malate synthase and a green algal glycolate dehydrogenase. All enzymes in the alternative pathway designs were directed to the chloroplast. RNA interference (RNAi) was also used to down-regulate a native chloroplast glycolate transporter in the photorespiratory pathway, thereby limiting metabolite flux through the native pathway. The three pathways were introduced with and without the transporter RNAi construct into tobacco, which is an ideal model field crop because it is easily transformed, has a short life cycle, produces large quantities of seed, and develops a robust canopy similar to that of other field crops.

## Results

Using a synthetic biology approach to vary promoter gene combinations, we generated a total of 17 construct designs of the three pathways with and without the transporter RNAi construct. Initial screens for photoprotection by alternative pathway function under high–photorespiratory stress conditions identified three to five independent transformants of each design for further analysis. Gene and protein expression analyses confirmed expression of the introduced genes and suppression of the native transporter in RNAi plants. In greenhouse screens, pathway 1 increased biomass by nearly 13%. Pathway 2 showed no benefit compared to wild type. Introduction of pathway 3 increased biomass by 18% without RNAi and 24% with RNAi, which were consistent with changes in photorespiratory metabolism and higher photosynthetic rates. Ultimately, field testing across two different growing seasons showed significant increases in biomass of pathway 3 plants with RNAi compared to WT of 20% in 2016 (P = 0.04) and by 24% in 2017 (P = 0.018). In addition, this pathway increased the light-use efficiency of photosynthesis by 17% in the field.

## Conclusion

Engineering more efficient photorespiratory pathways into tobacco while inhibiting the native pathway markedly increased both photosynthetic efficiency and vegetative biomass. We are optimistic that similar gains may be achieved and translated into increased yield in C_3_ grain crops because photorespiration is common to all C_3_ plants and higher photosynthetic rates under elevated CO_2_, which suppresses photorespiration and increases harvestable yield in C_3_ crops. ■

Population growth, increasing global affluence, and an expanding bioeconomy are conspiring to increase mid-century agricultural demand by 60 to 120% over 2005 levels, a challenge that current rates of crop productivity improvement averaging <2% per year cannot meet (1–3). In the 45 years after 1960, global crop productivity increased 135% from 1.84 to 3.96 metric tons per hectare (4). The increased use of pesticides, fertilizers and irrigation, and mechanization, along with the adoption of higher-yielding crop varieties that drove this remarkable global increase in productivity, are now largely optimized for major crops and are unlikely to generate sufficient yield increases to meet mid-century agricultural demand. However, photosynthetic efficiency remains standing as a determinant of yield potential with the improvement capacity to double crop productivity (1–3, 5, 6). In C_3_ crops such as wheat, rice, and soybeans, photorespiration reduces the photosynthetic conversion efficiency of light into biomass by 20 to 50%, with the largest losses occurring in hot dry climates where yield increases are sorely needed. Whereas ribulose-1,5-bisphosphate carboxylase-oxygenase (RuBisCO) carboxylates ribulose-1,5-bisphosphate (RuBP) during photosynthesis, the unproductive and energyintensive process of photorespiration results from oxygenation of RuBP by RuBisCO, which becomes more prevalent at higher temperatures and under drought conditions (6,7). Toxic by-products of the RuBisCO oxygenation reaction (2-phosphoglycolate and glycolate) and of the glycine decarboxylation reaction (ammonia) are recycled by photorespiration into nontoxic products but at the expense of energy and net loss of fixed carbon (6,7). Some photosynthetic algae, bacteria, and plants have evolved mechanisms to reduce the oxygenation reaction by RuBisCO via carbon-concentrating mechanisms (CCMs), including C4 photosynthesis (8,9), inspiring efforts to introduce CCMs into C_3_ plants (8–12). Here we have taken an alternative approach of introducing non-native and synthetic metabolic pathways to recycle the products of RuBisCO oxygenation more efficiently (13). Previously, two alternative photorespiratory pathways implemented in Arabidopsis improved photosynthesis and plant size in chamber and greenhouse experiments (14,15). These results inspired us to optimize these alternative photo-respiratory pathways in tobacco, a useful agricultural model crop, for field trials. Computer modeling of these alternative pathways revealed the importance of optimized expression of nonnative genes to achieve maximum flux through the alternative pathway and thus maximize the benefits for crop plants under field conditions (16). Additionally, we sought to minimize flux through the native photorespiratory pathway and maximize flux through the introduced pathways by inhibiting glycolate export from the chloroplast.

## Results

### Transgene assembly

**Fig. 1:**
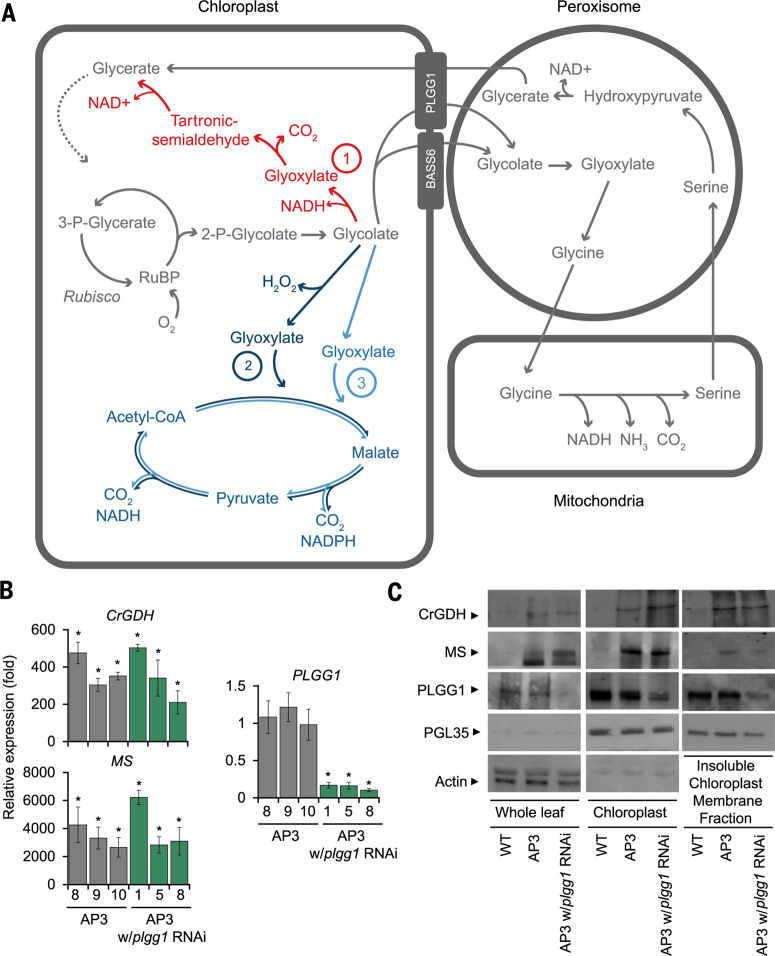
***Alternative photorespiratory pathways.*** (***A***) Model of three alternative photorespiration pathway designs. API (red) converts glycolate to glycerate using five genes from the E. coli glycolate pathway encoding the enzymes glycolate dehydrogenase, glyoxylate carboligase, and tartronic semialdehyde reductase. AP2 (dark blue) requires three introduced genes encoding glycolate oxidase, malate synthase, and catalase (to remove hydrogen peroxide generated by glycolate oxidase). AP3 (blue) relies on two introduced genes: Chlamydomonas reinhardtii glycolate dehydrogenase and Cucurbita maxima malate synthase. (**B**) qRT-PCR analysis of the two transgenes in AP3 and the target gene PLGG1 of the RNAi construct. Results for three independent transformation events are shown with (1, 5, and 8) and without (8, 9, and 10) PLGG1 RNAi. Error bars indicate SEM. * indicates statistical difference at P < 0.05 compared to WT based on one-way ANOVA. Actual P values are shown in supplementary data set 15. (**C**) Immunoblot analysis from whole leaves and isolated chloroplasts, including the insoluble membrane fraction, using custom antibodies raised against the indicated target genes, cytosolic marker actin, and chloroplast-specific marker platoglobulin 35 (PGL35). Five micrograms of protein was loaded per lane. Arrows indicate detected protein based on molecular weight. The kinetic properties of CrGDH, as well as numerous malate synthase enzymes, have been previously characterized (table S3) (17).

We transformed Nicotiana tabacum cv. Petite Havana (tobacco) with three different photores-piratory alternative pathway (AP) designs, expressing as many as seven genes in single constructs (Fig. 1A and table S1). Tobacco is an ideal model crop for these studies because of its completely sequenced genome, short life cycle (3 months from seed to seed), well established high-efficiency transformation protocols, and the ability to form a fully closed canopy like other crops in the field. The AP1 construct targets the five genes of the Escherichia coli glycolate oxidation pathway to the chloroplast (Fig. 1A) (14). AP2 includes Arabidopsis glycolate oxidase (GO) and Cucurbita maxima (pumpkin) malate synthase (MS), along with a catalase (CAT) from E. coli (Fig. 1A) (15). AP3 also contains C. maxima MS sequence but replaces the plant GO used in AP2 with Chlamydomonas reinhardtii glycolate dehydrogenase (CrGDH) to avoid hydrogen peroxide production when glycolate is converted to glyoxylate (Fig. 1A). With this modification, expression of E. coli CAT in the chloroplast is unnecessary (17). Using multigene constructs assembled from modular parts by Golden Gate cloning, we generated multiple promoter gene combinations and within-construct position effects to optimize AP performance. We generated five iterations of AP1, three iterations of AP2, and a single design of AP3 for testing (table S1). In addition to the expression of the AP genes, we designed a long hairpin RNA interference (RNAi) construct and added it to the library of multigene constructs to reduce the expression of the chloroplast glycolate-glycerate transporter PLGG1 with the goal of minimizing glycolate flux out of the chloroplast and into the native pathway (Fig. 1 and table S1) (18,19). In total, we successfully transformed 17 different constructs of the three AP designs into tobacco with and without the inclusion of an RNAi module targeting the PLGG1 transporter.

## Gene and protein analysis confirm chloroplast-localized transgene expression

Transgene expression analysis conducted on three independent transformants of each AP design selected for further analysis confirmed strong expression of the transgenes along with ~80% RNAi suppression of PLGG1 expression (Fig. 1B and fig. S1). Immunoblot analysis of whole-cell extract was normalized on the basis of total protein content and verified using antibodies against the RuBisCO large subunit and actin (fig. S2). Immunoblot analysis of isolated intact chloroplasts from AP3 plants (Fig. 1C) verified that the construct design of AP3 directs CrGDH and MS protein to the chloroplast and that RNAi suppresses expression of the PLGG1 transporter protein. The cytoplasmic marker protein actin was undetectable in the isolated chloroplast fraction, ensuring that the AP3 proteins in the chloroplast fraction was not a result of cytoplasmic contamination (Fig. 1C). Moreover, the chloroplast marker PGL35 was only faintly detectable in the whole-leaf extracts but was greatly enriched in the isolated chloroplast fraction (Fig. 1C). Whereas MS was also greatly enriched in the chloroplast fraction, CrGDH appeared to be much less enriched in this fraction (Fig. 1C). Glycolate dehydrogenases have been shown to be strongly associated with membranes in both chlorophytes and bacteria (20,21) and thus may have been inefficiently extracted from our chloroplast preparation (17). Isolation of the insoluble membrane fraction from the chloroplast extraction showed that a large fraction CrGDH in tobacco chloroplasts was associated with the membranes (Fig. 1C) and that CrGDH was enriched relative to PGL35 in the membrane fraction.

## AP plants are resistant to photorespiration stress

**Fig. 2:**
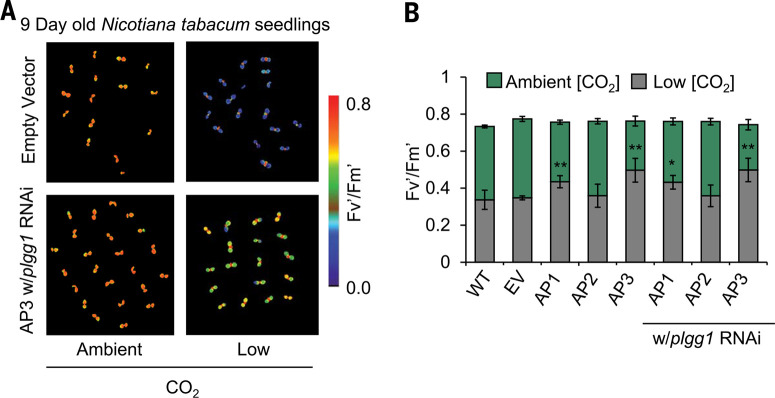
***AP plant lines are more photoprotective under photorespiration stress. (A*)** Representative photos of 9-day-old T2 transgenic tobacco lines during the chlorophyll fluorescence photoprotection screen for AP pathway function showing AP3 protecting photosystem II from photodamage under severe photorespiratory conditions. (**B**) Combined values of the three AP construct designs with and without RNAi targeting the glycolate-glycerate transporter PLGG1. Error bars indicate SEM. * indicates statistical difference compared to WT based on one-way ANOVA at *P* < 0.05, ** *P* < 0.001. Fv'/Fm' for individual lines is described in supplementary data set 1. Actual significant *P* values are shown in supplementary data set 15.

Following selection for construct expression by selectable marker screening [BASTA resistance (bar) gene added to all constructs] (table S1) and genotyping selection for single-insert homozygous transgenic plants, all independent constructs of the three AP designs were assessed for resistance to photorespiration stress in a high-throughput chlorophyll fluorescence assay. Photorespiratory mutants typically display impaired growth and photosynthesis when transferred from elevated CO_2_ concentrations ([CO_2_]) to ambient air, which is accompanied by the onset of photoinhibition that can be diagnosed by monitoring chlorophyll fluorescence (19, 22–24). We hypothesized that AP function would be photoprotective under high photorespiratory stress, thus protecting photosystem II operating efficiency (i.e., Fv′/Fm′) from photodamage (19, 22). Previously, this method of monitoring Fv′/Fm′ after illumination in low [CO_2_] enabled identification of photorespiration mutants that cause photoinhibition (19, 22, 24). Using this protocol to monitor AP function, we exposed thousands of single-insert homozygous T2 seedling plants to 24 hours of high light intensity (1200 µmol m−2 s−1) and very low [CO_2_] (1 to 38 µbar CO_2_) and then compared Fv′/Fm′ in the transformants with azygous wild-type (WT) and empty vector (EV) controls (fig. S3). Many independent transformants (66% of AP1, 54% of AP2, and 84% of AP3 plants) were significantly more photoprotective under this severe photorespiratory stress. Versions of AP1 and AP3 sustained 33 to 48% higher Fv′/Fm′ values compared to WT and EV controls (Fig. 2, A and B, and data set S1). Under ambient [CO_2_], there were no observed differences in Fv′/Fm′ between the AP and control lines. However, PLGG1 RNAi inhibition of glycolate efflux from the chloroplast reduced Fv′/Fm′ when these plants were shifted from elevated [CO_2_] to ambient (fig. S4). This photoinhibited phenotype of the PLGG1 RNAi plants was not only rescued by transgenic complementation with AP1 or AP3 constructs, but was also substantially more resistant to photoinhibition than WT and EV controls (Fig. 2B and dataset S1).

## AP plants show enhanced biomass accumulation in greenhouse growth studies

**Fig. 3:**
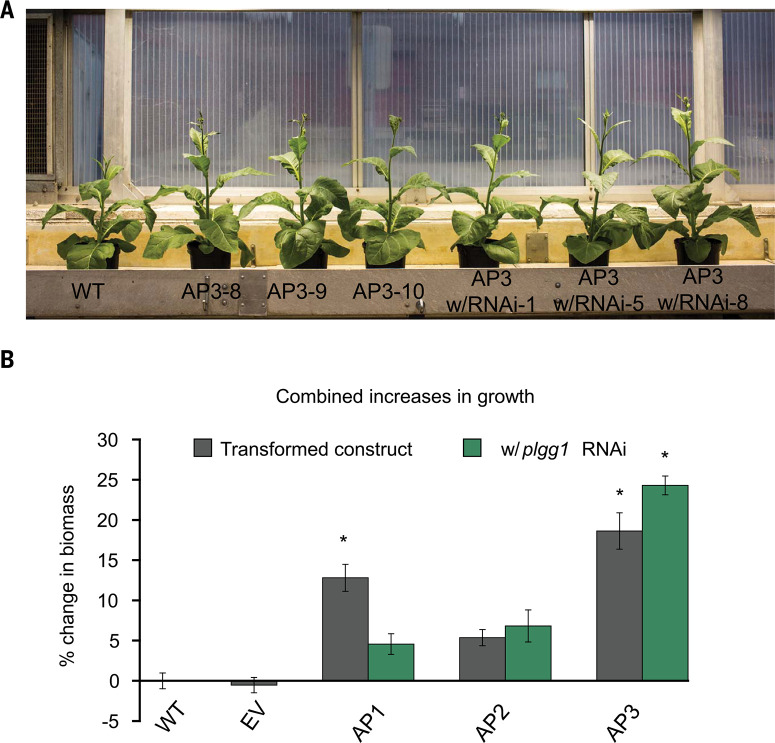
Photorespiration AP lines increase biomass under greenhouse conditions. (A) Photos of 6-week-old AP3 and WT plants grown in the greenhouse. Individual plant lines are indicated in the labels below the plant. (B) Percent difference in total dry weight biomass of the indicated combined plant lines. * indicate statistical difference based on one-way ANOVA. Error bars are SEM, n = 7 (plants measured), P < 0.05 values listed in data set 15.

Following the initial photoprotection screen and expression analysis, we determined the impact of the three APs on plant growth in greenhouse growth studies. Both the AP1 and AP3 designs significantly increased dry-weight biomass relative to the WT plants. Overall, AP1 plants increased dry weight biomass by 13%, but the benefit was lost when the PLGG1 RNAi module was present (Fig. 3B). AP2 introduction did not significantly alter dry weight (Fig. 3B). Three AP3 lines that sustained much higher Fv'/Fm' values (200-8,9,10) compared to WT and EV were taller (Fig. 3A) and showed the largest increases in biomass in greenhouse studies, with a 24% increase with and 18% increase without the PLGG1 RNAi module compared to WT (Fig. 3B). We also tested an AP3 line that had the same Fv'/Fm' as WT and EV (200-4), which showed no increase in biomass, and one line that had an intermediate Fv'/Fm' (200-6) that showed a small but statistically significant biomass increase in greenhouse studies (fig. S5, A and C). Transcript expression analysis of AP3 events 200-4 and 200-6 revealed that CrGDH and MS expression was greatly reduced compared to transgenic events 200-8,9,10 (fig. S5B).

## AP3 plants have an altered photorespiratory metabolite profile

**Fig. 4:**
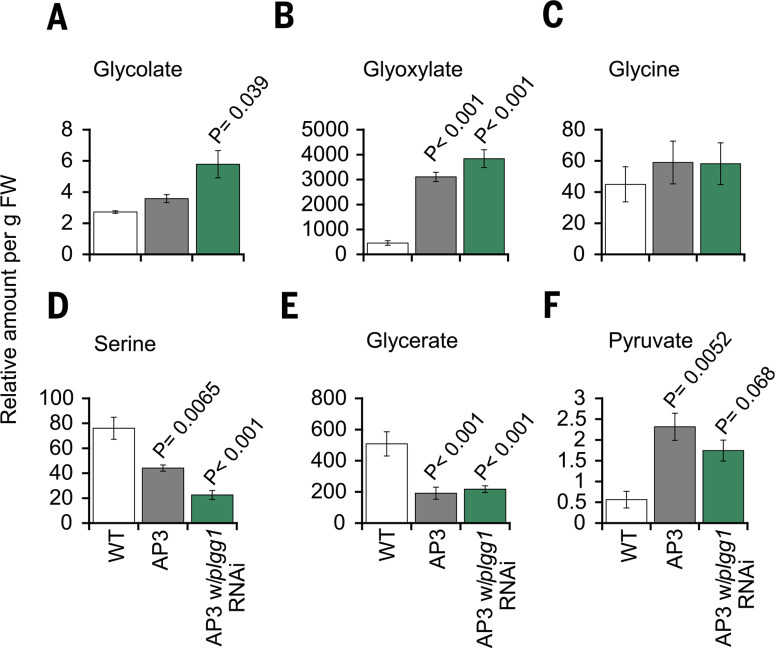
***Photorespiratory and AP3 metabolic intermediates.* (*A*** to***F*)** Relative amount of the indicated metabolite detected from ~40 mg of leaf tissue (fresh weight; FW) sampled in the late morning. Metabolite concentrations were reported as concentrations relative to the internal standard, which is the target compound peak area divided by peak area of hentriacontanoic acid: N (relative concentration) = Xi (target compound peak area) * X^-1^IS (peak area of hentriacontanoic acid) per gram fresh weight. Error bars indicate SEM, n = 4 leaf samples. Statistical differences between AP3 designs and WT based on one-way ANOVA, with P values indicated. All P values are listed in dataset 15.

We further investigated the AP3 plants that showed the greatest growth stimulation and gene expression to determine the effect of AP3 enzymes on the leaf photorespiratory metabolite profile. We performed gas chromatography followed by mass spectrometry on leaf samples from greenhouse-grown WT and AP3 plants to analyze the photorespiratory intermediates glycolate, glyoxylate, glycine, and serine and the AP3-specific intermediate pyruvate (Fig. 4). AP3 introduction with and without the RNAi module increased glyoxylate and pyruvate concentration compared to WT, suggesting altered native photorespiration and possibly flux through the alternative pathway (Fig. 4, B and F). AP3 plants with and without the RNAi module also had decreased concentrations of the photorespiratory intermediates serine, for which photorespiration is a major source (25), and glycerate, possibly due to a diversion of carbon away from the native photorespiratory pathway (Fig. 4, D and E). Glycine concentrations were similar in AP3 and WT plants (Fig. 4C). AP3 with the RNAi module targeting the glycolate-glycerate transporter PLGG1 had increased glycolate accumulation compared to WT in a manner similar to the Arabidopsis T-DNA insertion mutant plgg1-1 (Fig. 4A) (18,19).

## AP3 plants exhibit increased photosynthetic rate and chloroplast [CO_2_]

**Fig. 5:**
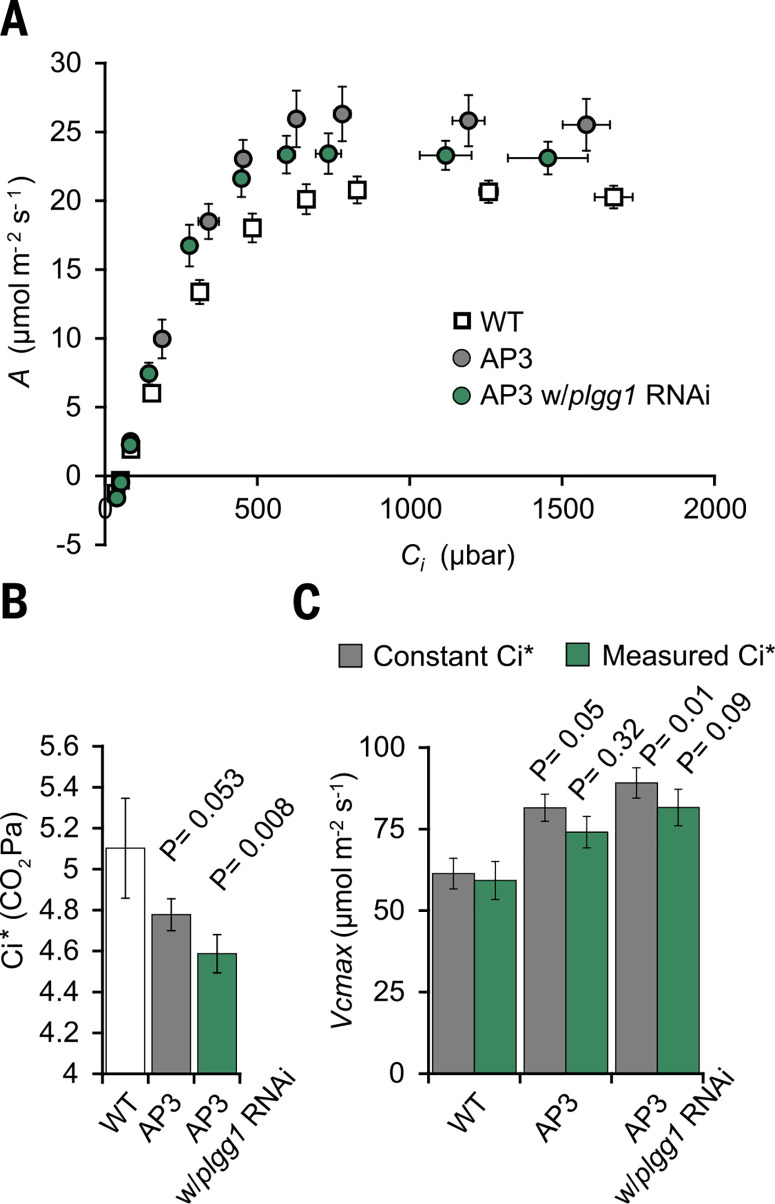
***Photosynthetic efficiency of greenhouse-grown plants.*** Data are the combined result of three independent transformants (hereafter referred to as combined) with and without PLGG1 RNAi. (A) CO_2_ assimilation based on intercellular [CO_2_] (C_i_). (**B**) Combined apparent CO_2_ compensation point: C_i_* calculated using the common intercept method and slope regression (29). (**C**) Combined maximum rate of RuBisCO carboxylation (*V*_cmax_). *V*_cmax_ values are presented at 25°C and modeled from photosynthetic response under changing CO_2_ concentration. Gray bars indicate constant C_i_*; green bars indicate derived values based on measured C_i_*. Error bars indicate SEM. P values for statistical comparison to WT based on one-way ANOVA are given.

To test if altered photorespiration due to introduction of the AP3 design in plants affects rates of photosynthesis, we compared CO_2_ assimilation rates *(*A*)* as a function of intercellular CO_2_ concentrations (C_i_) under saturating light in AP3 and WT plants. AP3 plant lines with and without the PLGG1 RNAi module had increased rates of photosynthesis compared to WT (Fig. 5A). Modeling of the A/C*_i_* curves showed increases in the maximum rate of RuBisCO carboxylation (*V*_cmax_) visualized in the initial slope of the A/C*_i_* curve in AP3 lines (Fig. 5, A and C). We observed no statistical differences in the maximum rate of electron transport (*J*_max_) in any AP design (fig. S6). Increases in *V*_cmax_, which is a property of RuBisCO enzymatic activity, could be due to increased RuBisCO protein content or increased availability of CO_2_ as a substrate for RuBisCO. Immunoblot analysis shows no difference in RuBisCO content on a per microgram protein basis (fig. S2), suggesting that the observed difference is based on increased availability of CO_2_ at the site of carboxylation in the chloroplast. Increases in CO_2_ availability for RuBisCO carboxylation could arise from increased mesophyll conductance (*g*_m_; i.e., the diffusion of CO_2_ into mesophyll cell chloroplasts) or from the direct release of photorespiratory CO_2_ in the chloroplast by the decarboxylation of malate and pyruvate in the plastid (Fig. 1A), both of which would result in observed increase in *V*_cmax_ determined from *A*/*C*_i_ curves (*26*). There is no apparent reason to expect that the introduction of these alternative pathways would decrease the resistance for the movement of CO_2_ from the mesophyll intercellular air space to the chloroplast stroma or from the mitochondria to the chloroplast stroma. However, an increase in gm contributing to increased CO_2_ availability within the AP3 plant chloroplast is difficult to rule out, largely because modeling of gm requires knowledge of, or assumptions about, the conductance of CO_2_ released from the mitochondria during the conversion of glycine to serine to the chloroplast, which is directly affected by the introduction of the alternative pathway.

Theory predicts that the release of photorespiratory CO_2_ in the chloroplast by the AP pathways, instead of in themitochondria through the native photorespiratory pathway, would lower *C*_i_*, the intercellular [CO_2_] at which the chloroplast [CO_2_] reaches F020Γ*, the [CO_2_] in the chloroplast at which the rates of RuBisCO oxygenation and carboxylation are equal (*27*–*29*). To determine C_i_*, we measured the internal [CO_2_] at which CO_2_ response curves measured at different subsaturating illumination intensities intersect (*29*). AP3 plants with the RNAi module targeting PLGG1 showed a significant reduction of 10% in *C*_i_* and AP3 plants without the RNAi module showed a significant reduction of 6.4% in C_i_* compared to WT(Fig. 5B). The observed decreases in *C*i*, coupled with unaltered respiration (fig. S7), are consistent with elevated chloroplastic [CO_2_] due to decarboxylation of malate and pyruvate within the introduced pathway (Figs. 1A and 5B and fig. S6), which would also explain the significantly higher values of *V*_cmax_ in AP3 plants compared to WT plants (Fig. 5, A and C, and fig. S6). Accounting for the observed C_i_* in *A*/*C*_i_ curve analysis reduces the apparent change in *V*_cmax_ further indicating that the difference in *V*_cmax_ was not due to changes in RuBisCO content or activity but rather by increased chloroplastic [CO_2_] (Fig. 5C).

## AP plants show increased photosynthetic rates, quantum efficiency, and biomass accumulation in replicated field trials

In the 2016 growing season, we tested four independent events of AP1, two independent events of AP2, and five independent transformation events of AP3, along with two WT and two EV controls in the field, using a randomized single block design experiment (fig. S8). Biomass increased by 16% in AP1 lines and 10% in one of the AP2 lines tested (fig. S9). The three AP3 lines that showed the largest biomass increases in the greenhouse consistently showed the largest increases in dry-weight biomass, with total biomass increasing by as much as 23% relative to WT (fig. S9). Independent AP3 events 200-4 and 200-6, in which CrGDH and MS expression was significantly lower compared to other transgenic events (fig. S5B) and showed less or no improvement in greenhouse biomass (fig. S5C), also showed no increases in total biomass in the 2016 field season (fig. S9). We anticipated, owing to their lower energetic requirements, that the AP pathways would improve the maximum quantum efficiency of net CO_2_ assimilation (Fa) relative to the native pathway. Fa increased in lines of all AP pathways, in many cases by >20%, including those containing the RNAi module targeting the PLGG1 transporter (fig. S10) but not in AP3 events 200-4 and 200-6 (fig. S11A). The high-biomass-producing AP3 plant lines exhibited an increased light-saturated rate of assimilation compared to WT, to several AP1 lines, and to all AP2 plant lines (fig. S10C) and to AP3 events 200-4 and 200-6 (fig. S11).

**Fig. 6:**
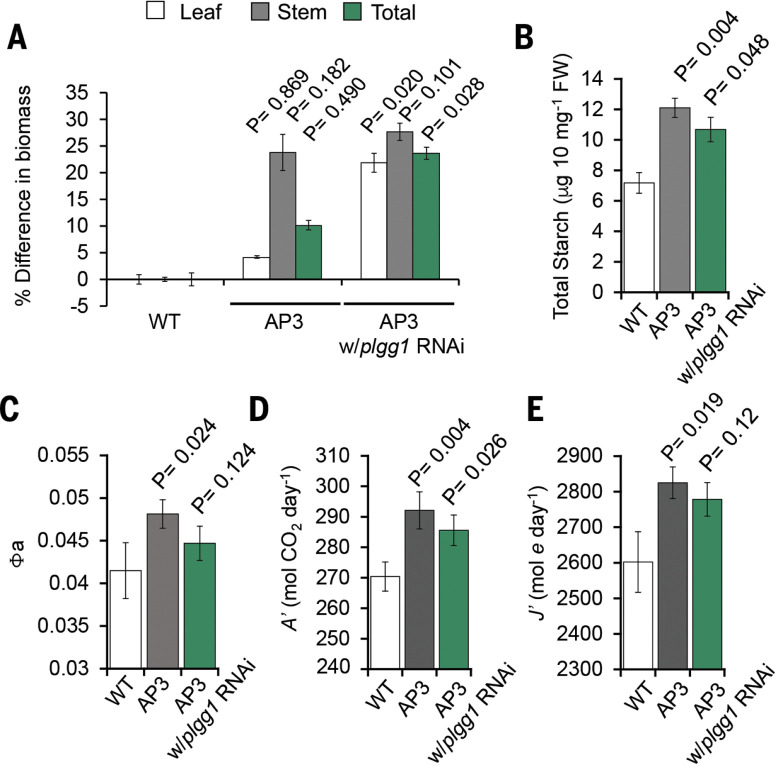
***Plant productivity and photosynthetic performance in 2017 field trials.*** (***A***) Percent difference from WT for stem, leaf, and total biomass of AP3 with and without the PLGG1 RNAi module. Data are the combined result of three independent transformants with and without PLGG1 RNAi. (**B**) Total combined accumulated leaf starch for indicated lines extracted from 10 mg of fresh weight leaf material. (**C**) Combined apparent quantum efficiency of photosynthesis (Fa) determined by linear regression of assimilation based on available light-response curves. (**D**) Combined accumulated assimilation of CO_2_ (A') based on diurnal analysis of photosynthesis. (**E**) Combined accumulated electrons used in electron transport determined from assimilation based on diurnal photosynthesis. Error bars indicate SD, and P values are indicated based on two-way ANOVA.

To validate the 2016 field results and improve the statistical power of comparisons with AP3 plants under agricultural conditions, we tested five randomized replicate blocks of three AP3 independent transformed lines with and without the RNAi module targeting PLGG1 in comparison to WT during the 2017 growing season (fig. S12). The AP3 plant lines showed a 17% difference in 2016, but in 2017 the 10% increase in dry-weight biomass (4% leaf, 24% stem) was not significant. The inclusion of the PLGG1 RNAi module in AP3 designs further increased leaf dry biomass to 22%, stem dry biomass to 28%, and total dry biomass to 24% compared to WT (Fig. 6A). That AP3 plant lines with the RNAi module showed a significant leaf and total dry weight biomass increase (17% and 13%, respectively) over the AP3-only plants supports our hypothesis that forcing greater glycolate flux through the synthetic pathway by inhibiting flux through the native photorespiratory pathway drove the increased productivity. Total mid-day starch content in AP3 plants increased by ~70% and in AP3 with PLGG1 RNAi by ~40% compared to the WT control (Fig. 6B). The apparent quantum efficiency of photosynthesis increased in both AP3 plant pathways; by 7% with and 17% without PLGG1 RNAi for the 2017 field season (Fig. 6C and fig. S13). Because plants with both AP3 designs exhibited increases in the quantum efficiency of photosynthesis and decreases in Ci*, we hypothesized that total daily net carbon gain through photosynthesis would be higher compared to WT, resulting in the observed increases in biomass over the growing season (Figs. 5B and 6, A and C). Indeed, modeled daily net carbon gain from measurements of photosynthesis over a diurnal time course in plants containing AP3 showed an increase of 5 to 8% in CO_2_ assimilation (A') and increases in electron use in photosynthesis (J') compared to WT (Fig. 6, D and E).

## Discussion

We showed that installing synthetic glycolate metabolic pathways into tobacco chloroplasts drove large increases in biomass accumulation in both greenhouse conditions and in the field under agricultural conditions. Because AP3 plants exhibited the greatest growth stimulation, we selected this pathway for more in-depth characterization. In summary, the AP3 transgene products CrGDH and MS localized to the chloroplast (Fig. 1C). Evidence that these transgene products function in the chloroplast to catalyze the reactions depicted in Fig. 1 include the stimulation of the rate of photosynthesis (Fig. 5) and improvement of photosynthetic quantum yield (Fig. 6 and fig. S10), the lowering of Ci* (Fig. 5) and increase in the initial slope of an A/Ci relationship (Fig. 5) that both indicate increased [CO_2_] in the chloroplast, and the altered photorespiratory metabolite profile (Fig. 4). In addition, the direct role of this pathway in chloroplast glycolate metabolism is supported by its ability to prevent photoinhibition and rescue the reduced growth phenotype of PLGG1 RNAi tobacco lines (fig. S4). Moreover, AP3 lines that contain the full transgene construct but with reduced transgene expression showed less, or no improvement in greenhouse (fig. S5C) or field biomass (fig. S9), and Fa values similar to WT (fig. S11) provide evidence that the amount of expression of the introduced alternative photorespiratory pathway drove the extent of improved growth and increased photosynthetic efficiency.

Of the two alternative pathways to photorespiration that inspired our designs (13,14), AP2 showed limited improvements in plant productivity, and 24% of the independent transgenic AP2 lines resulted in stunted growth and yellow leaves (fig. S14C). The AP1 design improved productivity in tobacco, but the enhancement associated with AP1 was eliminated in both greenhouse and field settings when the PLGG1 RNAi module was added (Fig. 3 and fig. S9). Modeling (16) predicted that directing the complete flux of glycolate through the AP1 pathway by inhibiting glycolate export from the chloroplast would result in the largest increase in energy savings, photosynthetic efficiency, and growth among all designs. Elimination of the AP1 enhancements by the PLGG1 RNAi module implies that this introduced pathway may not have had sufficient kinetic capacity to handle the full glycolate flux under high rates of RuBisCO oxygenation. Further optimization of expression of AP1 genes and/or use of AP1 genes of different origins and kinetic properties may lead to achieving the full benefits that modeling predicts for this design. The AP3 design containing C. maxima MS and CrGDH reliably increased plant biomass and improved photosynthetic efficiency (Figs. 3,5, and 6), and the phenotype is dependent on the level of expression of the transgenes in the independent transformation events (fig. S5). The inclusion of an RNAi module to reduce expression of the PLGG1 chloroplast glycolate-glycerate transporter in numerous independent transformant plant lines increased postharvest dry-weight biomass compared to AP3 introduction alone by 13% (P = 0.0805) (Figs. 3 and 6A and fig. S13). Without an alternative photorespiration pathway in place, inhibition of PLGG1 expression by RNAi decreased plant growth and led to photoinhibition (i.e., reduced Fv'/Fm') when these plants were transferred from elevated [CO_2_] to ambient air (fig. S4), as was reported previously for the plgg1-1 T-DNA knockout line in Arabidopsis (23). Thus, the genetic complementation of the low-growth photoinhibited phenotype and the significant increase in biomass in AP3 lines with RNAi over AP3 alone are consistent with the expected benefit of directing a greater proportion of the glycolate flux through the synthetic pathway in the chloroplast and away from the native photorespiratory pathway outside of the chloroplast (18,19). Indeed, forcing greater glycolate flux through the synthetic pathway by inhibiting glycolate transport out of the chloroplast through PLGG1 into the native photorespiratory pathway resulted in growth stimulation in field experiments between 19 and 37% (with an average effect size of 24%, P = 0.028) for the AP3 plants with RNAi. The glycolate-glycerate exchange transporter PLGG1 works in tandem with a second glycolate exporter BASS6 to stoichiometrically balance the export of two glycolate molecules with the import of one glycerate molecule during photorespiration (18). Thus, targeting the expression of both transporters may further test AP3 kinetic capacity and may drive even greater growth stimulation. Recognizing that these alternative pathways are intervening in the central metabolism of photosynthetic cells, it will be important to validate the biochemistry that is occurring as AP pathway intermediates may well have destinations that are different from those depicted in Fig. 1A.

Although inhibiting photorespiration under normal oxygen-containing atmospheres invariably results in inhibited photosynthesis and growth (7), some evidence indicates that stimulating photorespiratory flux can enhance photosynthetic rate and plant growth. Overexpression of the H-protein in the glycine decarboxylase complex or overexpression of plant glycolate oxidase (GO) can lead to increased photosynthesis and biomass production (30,31). In both of these reports, the overexpression of these photorespiration genes was accompanied by an increase in stomatal conductance that itself would be expected to increase photosynthesis and growth under water-replete conditions. Conversely, four different photorespiration mutants (pglp1, shm1, hpr1, and glyk1) partially lost stomatal responsiveness to altered CO_2_ availability, possibly indicating that alternative pathways could influence plant adaptation through stomatal signaling (32). We saw no statistical differences in stomatal conductance (fig. S15, A and B) or the expression of GO (fig. S15C) in AP3 tobacco plants, indicating that neither of these contributed to the stimulations observed in AP3 plant lines. Whether the installation of these alternative pathways may affect global changes in the transcriptome and the proteome and if that may have secondary impacts on plant growth outside of changes to primary metabolism remain to be determined. Energy demand calculations suggest that AP3 would consume more adenosine 5'-triphosphate than native photorespiration, similar to AP2 (33). It is likely that CrGDH uses the electron transport chain as an electron acceptor (17), and the decarboxylation of malate and pyruvate generate reducing equivalents (Fig. 1). However, the global effect of AP3 and PLGG1 repression on energy balance, as well as the possible fate of intermediates from AP3 in sucrose synthesis or the tricarboxylic acid cycle, will need to be assessed (17, 33).

Tobacco was selected for these proof-of-concept experiments not only for its ease of genetic transformation but also because it is an ideal model crop that is robust in the field, forms a fully closed canopy, and produces large quantities of seed, circumventing the need for numerous seed amplification generations, further accelerating the timeline to field testing. The photorespiratory mechanism is common to all C_3_ plants, although energetic costs and yield reductions depend on species-specific kinetic properties of RuBisCO, as well as the temperature and [CO_2_] under which the crop is growing. Previous work has demonstrated that alternative photorespiration pathways show a benefit to crop plants Camelina sativa (34) and potato (35) in greenhouse and chamber experiments, but it remains to be seen whether the increase in vegetative biomass that we observed in tobacco with AP3 in the field can be translated into increased seed or tuber production in crops such as soybean, cowpea, and potato. In greenhouse studies, only one AP3 line containing the PLGG1 RNAi module showed a significant increase in total seed weight (fig. S13E), but seed is not a major sink in tobacco as it is in grain crops. However, because increased photosynthetic efficiency due to the suppression of photorespiration in C_3_ crops grown in elevated [CO_2_] results in increased seed yield (5,36), we are optimistic that use of alternative metabolic pathways to photorespiration will also lead to increases in seed yield. Indeed, in this work, the observed stimulation of whole-plant biomass production was much larger than the stimulation of photosynthesis on a leaf area basis (5 to 8% increase in CO_2_ assimilation resulting in 10 to 24% increase in dry-weight biomass; compare Fig. 6A with Fig. 6, D and E), showing the benefit of compound interest from creating greater leaf area earlier in the growth cycle.

## Materials and Methods

### Plant genetic transformation

Nicotiana tabacum cv. Petite Havana was genetically transformed using Agrobacterium tumefaciens strain C58C1-mediated transformation (37). The 17 binary plasmids used in this study were assembled as described and listed in table S1 (19). AP1 genes originated from E. coli (14). AP2 genes originated from Arabidopsis thaliana (glycolate oxidase) and Cucurbita maxima (malate synthase) and E. coli (catalase) sources as described (15, 38). AP3 genes originated from Chlamydomonas reinhardtii for glycolate dehydrogenase and as described for AP2 for C. maxima malate synthase (15,17). Targeting to the chloroplast was designed by the addition of either the Arabidopsis RuBisCO small subunit (RbcS) or phosphoglucomutase transit peptide sequence added to the N terminus of the gene constructs. The RNAi module that targets the plastidic glycolate-glycerate transporter PLGG1 was designed using 300 base pairs of exon sequence derived from the Sol genomics network (https://solgenomics.net). All binary plasmids contained the BASTA resistance (bar) gene as a selectable marker for plant transformation. A minimum of 10 independent T0 transformations were generated to produce T progeny. T-DNA copy number was determined on T plants through quantitative reverse transcription-quantitative polymerase chain reaction (qRT-PCR) analysis (iDNA Genetics, Norwich UK) (dataset 17) (39). From these results, a minimum of five independent transformation events were selected to self and produce T2 progeny. Copy-number analysis was repeated to verify single insert homozygous lines for each transformation event. Nonsingle insert lines were not further characterized (for a representative timeline of characterization of AP3 lines see dataset 20). All WT controls used in this study were azygous plants, which have been through the transformation protocol but lost the construct including the selectable marker resistance during segregation.

### Chlorophyll fluorescence measurements

Tobacco T2 seeds were germinated under ambient air conditions on Murashige and Skoog (MS) plates with essential vitamins in a controlled environment chamber (Environmental Growth Chambers, Chagrin Falls, Ohio, USA) with 14 hours day (25°C)/10 hours night (22°C) and light intensity of 500 µmol m–2 s –1. Eight days after germination, seedling plates were transferred to a custom assembled low-[CO_2_] chamber inside the controlled environment growth chamber (fig. S1). The light levels were increased to 1200 µmol m–2 s –1 for 24 hours and [CO_2_] was maintained below 38 mbar (fig. S1). For PLGG1 RNAi-only plants, which have strongly depressed photorespiratory capacity, Ta lines were germinated on soil under elevated [CO_2_] conditions for 9 days and transferred to ambient air for 3 days prior to screening. Fv'/Fm' was determined on each plate using the CF Imager Technologica (http://www.technologica.co.uk/). Maximum flash intensity was 6800 µmol m–2 s–1 for 800 ms. Image values were obtained for each individual plant by detecting colonies within the fluorimager software program defining each position as described (19,22,40).

### Gene expression and protein detection

Plants were grown under greenhouse or field conditions as described below. Five leaf discs were harvested from three plants per line (2.9 cm2, ~100 mg). RNA and protein were extracted from the same leaf samples using the NucleoSpin RNA/ Protein kit (Macherey-Nagel GmbH & Co.KG, Duren, Germany). cDNA was generated from extracted RNA using the Quantinova reverse transcriptase kit (QIAGEN, USA). A minimum of three biological replicates, including three technical replicates each, were performed for all samples. Gene expression was analyzed using a Bio-Rad CFX connect real-time PCR system (Bio-Rad Laboratories, USA). Relative changes in transcript levels were determined using the AACt method with primers directed toward the transgene transcripts and the L25 gene as a standard control gene (41). cDNA was amplified using a SSO advanced SYBR green master mix (Bio-Rad), and primer sequences are described in table S2.

Total protein from AP3 was extracted using the Nucleospin protein/RNA kit described above or from frozen leaf material ground in liquid nitrogen, resuspended in lysis buffer [50 mM HEPES (pH 7.6), 300 mM sucrose, 2 mM MgCl_2_] plus plant protease inhibitor cocktail (Sigma-Aldrich). Protein was quantified using the protein quantification assay (Macherey-Nagel GmbH & Co. KG, Duren, Germany). Unless indicated otherwise, 5 mg of protein was loaded per lane and separated by 10% SDS-polyacrylamide electrophoresis (SDS-PAGE). PAGE gels were transferred to polyvinylidene difluoride (PVDF) membranes (Immobilon-P, Millipore, USA) using a Bio-Rad semi-dry transfer system or the Bio-Rad TransBlot turbo system. After blocking in a 6% milk TBS solution, membranes were incubated with custom antibodies raised against the malate synthase (MS) and PLGG1 (Agrisera, Vannas, Sweden) and glycolate dehydrogenase (GDH) (Genscript, USA). As a protein loading control, antibodies raised against the large subunit of RuBisCO (RbcL) and actin were used (Agrisera, Vannas, Sweden). After subsequent washing and incubation with anti-rabbit secondary antibody (Bio-Rad, USA), chemiluminescence was detected with the Image-Quant LAS4010 scanner (GE Healthcare Life Sciences, Pittsburgh, USA).

Chloroplasts were isolated in a manner similar to that described (19), with tobacco-specific modifications following (42). Leaf tissue was collected from 6-week-old WT and AP3 plants, briefly homogenized in extraction buffer [50 mM MES-NaOH (pH 6.1), 0.33 M sorbitol, 2 mM EDTA, 2 mM MgCl_2_,1 mM MgCl_2_, 20 mM NaCl, 2 mM isoascorbic acid, and 1% polyvinypyrrolidone-40], filtered through three layers of Miracloth (Calbiochem), and centrifuged at 4°C for 4 min at 2500g to pellet chloroplasts. Pelleted chloroplasts were resuspended in 5 ml of buffer [50 mM HEPES-NaOH (pH 6.8), 0.33 M sorbitol, 2 mM EDTA, 2 mM MgCl_2_,1 mM MgCl_2_, 5 mM isoascorbic acid, 1 mM sodium pyrophosphate, 5 mM glutathione] using a fine paintbrush, applied to a 20-ml Percoll density gradient [top to bottom: 40% (v/v) and 90% (v/v) Percoll in resuspension buffer], and centrifuged at 4°C for 30 min at 2500g. Intact chloroplasts accumulated at the 40 to 90% interface and were removed by aspiration, washed twice in 10 volumes of resuspension buffer, and collected by centrifugation for 10 min at 2500 g.

Plastid proteins were extracted by lysing the chloroplasts in a hypotonic buffer [10 mM Tricine-NaOH (pH 8.0), 1% (v/v) plant protease inhibitor cocktail (Sigma-Aldrich), and 5 mM dithiothreitol (DTT)], followed by two freeze-thaw cycles. Insoluble membrane fractions from the chloroplast isolation were isolated by centrifugation at 10,000 g for 5 min. The pellet was resuspended in 2x SDS sample buffer plus 10% DTT, then briefly sonicated. The membrane fraction proteins were then precipitated using ice-cold acetone. After centrifugation (10,000 g for 5 min), the acetone was removed, and the pellet was air dried. The protein pellet was then resuspended in SDS sample buffer plus 10% DTT. Protein concentration was then determined using a total protein quantification kit (Macherey-Nagel GmbH & Co.KG, Duren, Germany).

### Photorespiratory metabolite analysis

Metabolite analysis was performed as described (19). Briefly, ~40 mg of fresh leaf tissue was harvested from 6-week-old greenhouse-grown plants taken late morning (~10:00 to 11:00 a.m.) and flash frozen in liquid nitrogen. Leaf material was crushed using a genogrinder (Biospec products) and extracted in 100% ice-cold methanol. Samples were then submitted to the Metabolomics Center, Roy J. Carver Biotechnology Center, University of Illinois at Urbana-Champaign and processed as described (19). All known artificial peaks were identified and removed. To allow comparison among samples, all data were normalized to the internal standard in each chromatogram and the sample fresh weight. The spectra of all chromatogram peaks were evaluated using the AMDIS 2.71 program (NIST). Metabolite concentrations were reported as concentrations relative to the internal standard, which was the target compound peak area divided by peak area of hentriacontanoic acid: Ni (relative concentration) = Xi (target compound peak area) * X–1IS (peak area of hentriacontanoic acid) per gram fresh weight. The instrument variability was within the standard acceptance limit of 5%.

### Growth analysis (greenhouse)

Homozygous single-insert T2 seeds were germinated on LC1 Sunshine mix (Sun Gro 202 Horticulture, Agawam, MA, USA). Ten days after germination, seedlings were transferred to 4L pots (400C, Hummert International, Earth City, MO, USA) with LC1 Sunshine mix supplemented with slow-release fertilizer (Osmocote Plus 15/9/12, The Scotts Company LLC, Marysville, OH, USA). Pots were randomized within the greenhouse and positions were changed before each watering approximately every 4 to 5 days. Greenhouse growth conditions are tabulated in supplementary dataset 12. Aboveground biomass was harvested and dried for 2 weeks to attain constant weight, and dry weights determined for stem and leaf fractions. Stem fractions included reproductive material developed at time of final harvest.

### Field experiments

In 2016, five independent transformation events of AP3, four events of AP1, and two independent transformations of AP2, with two wild type (WT) and two empty vector (EV) controls, were planted in a randomized single block design. Homozygous single-insert T2 seeds were germinated in pots containing soil mix (Sun Gro 202 Horticulture, Agawam, MA, USA) on 14 May 2016 and grown for 7 days before transferal to floating trays as described (43). Plants were transplanted at the University of Illinois Energy Farm field station (40.11°N, 88.21°W, Urbana, IL, USA) on 6 June 2016 after the field was prepared as described (43). Each plot consisted of 6 ⨯ 6 plants spaced 30 cm apart (fig. S8). The internal 16 plants per plot were the indicated transgenic plant lines surrounded by a border of 20 WT plants. An additional two-row border of WT plants surrounded the full experiment that consisted of 26 plots. Watering was provided as needed from six water towers placed within the experiment. Weather data, including light intensity, air temperature, and precipitation, were measured for the 2016 field season as described (43) (supplementary data set 13).

Apparent quantum efficiency of photosynthesis (Φa) and the light-saturated rate of photosynthetic CO_2_ assimilation at ambient (400 µbar) and low (100 µbar) [CO_2_] were measured on the youngest fully expanded leaf 14 to 20 days after transplanting in the field. Φa was determined from assimilation measurements in response to light levels at the indicated [CO_2_]. Gas exchange measurements were performed using Li-Cor 6400XT instruments with a 2-cm2 fluorescence measuring cuvette for which chamber leaks were corrected as outlined in the manual (LI-COR Biosciences, Lincoln, NE, USA). Measurements of CO_2_ assimilation were conducted at incidental light intensities of 1200, 380,120, 65, 40, 30, 25,18, and 10 µmol m–2 s–1, and absorbed light was calculated using an integrating sphere (Ocean-Optics, Largo, FL, USA) (23). Assimilation was recorded after a minimum of 120 s at each light level. Fa was calculated from the slope of the initial linear response of CO_2_ assimilation at low light levels. The saturating rate of assimilation (Asat) was determined at 1200 µmol m–2 s–1 light intensity at the indicated [CO_2_]. Leaf and stem biomass were determined for 16 plants per plot at 7 weeks post planting. Aboveground biomass was harvested and separated into leaf and stem fractions. Plant material was dried at 65°C to constant weight for a minimum of 2 weeks prior to biomass measurements.

To increase the statistical power of the field experiment, the 2017 growing season focused on six independent transgenic AP3 lines. The field design consisted of five replicate blocks with seven randomized 6 × 6 plants plots per block (fig. S11). The central 16 plants were the AP3 transgenic line or WT surrounded by a WT border. The entire 35 plot-area was surrounded by an additional row of WT as a border. Singleinsert homozygous T2 lines generated from the same harvest time were sown on LC1 Sunshine mix and germinated for 7 days. After 7 days, seedlings were transplanted to floating trays as described above. Fourteen days after transplant to floating trays, plants were transplanted at the Energy Farm field station at the University of Illinois, Urbana, IL, USA, on 21June 2017. Watering was provided as needed using parallel drip irrigation. Photosynthesis measurements to determine Fa were performed 2 to 5 July, 2017, and Fa was measured as described above. Measurements of CO_2_ assimilation in response to light began predawn and were conducted at light intensities of 0, 10, 18, 25, 30, 40, 65, 120, 380, 1200, and 2000 µmol mol–1. Diurnal measurements of photosynthesis were performed starting pre-dawn on 14 July 2017 and measured every 2 hours on two plants per plot per block. Light levels and chamber temperature was set to ambient values based on incoming light levels using a PAR sensor on the Li-Cor 6400XT and a built-in temperature sensor. Reference [CO_2_] was maintained at 400 mbar. Diurnal measurements were continued until after dusk. At 49 days post-germination, eight plants per plot were harvested from all five replicate blocks. Aboveground biomass was separated into leaf and stem fractions and dried in a drying oven for 2 weeks to constant weight before biomass measurements. For starch analysis, 10 mg of leaf material was collected on 14 July, frozen in liquid nitrogen, and stored at –80°C. Starch was assayed using the Enzychrom starch assay kit (Bioassay Systems, Hayward, CA, USA). Colorimetric measurements were performed on a Biotek Synergy HT plate reader (Biotek Winooski, VT, USA).

### Photosynthetic CO_2_ response

Photosynthetic compensation point (Ci*) measurements were performed using a Li-Cor 6800 (Li-Cor Biosciences) equipped with a fluorescence chamber. Ci* was determined using the common intersection method by measuring the CO_2_ response of photosynthesis under various subsaturating irradiances (29, 44, 45). The common intersection was determined using slope-intercept regression to produce more accurate and consistent values of Ci* (29). Plants were acclimated under 250 µmol m–2 s–1 light at 150 µbar CO_2_ until photosynthesis reached steady state and then measured at 150,120, 90, 70, 50, and 30 µbar CO_2_ under light intensities of 250,165,120, 80, and 50 µmol m–2 s–1. The x-intersection point was converted to Ci* according to (29).

To determine the net photosynthetic assimilation rate from a CO_2_ dose response, the fifth leaf from the base of 7-week-old N. tabacum plants was measured using a Li-Cor 6800 infrared gas analyzer (Li-Cor Biosciences, Lincoln, NE, USA) with leaf temperature controlled at 25°C and light intensity set at 1500 µmol m–2 s–1. Leaves were acclimated at a [CO_2_] of 400 µbar to achieve a steady-state rate of assimilation. The [CO_2_] of the response curve was set at 400, 200,100, 50, 30, 400, 600, 800, 1000, 1500, 2000 µbar, and measurements were taken when assimilation reached a steady state rate. To determine the maximum rate of carboxylation (Vcmax) and maximum electron transport rate (Jmax), a model for leaf photosynthesis with temperature corrections was used assuming a mesophyll conductance of 0.57 mol–2 s–1 bar–1 (46), then adjusted using the determined value of Ci*.

### Statistical analysis

Statistical analysis was performed using Origin Pro 2016 (version 9.3.226, Origin Lab Corporation Northampton, MA, USA) or R (version 3.4.2 https://www.R-project.org/). For Fv'/Fm' measurements, each plate contained a minimum of 10 seedlings and the data shown reflect the averaged values. Significance was evaluated by one way analysis of variance (ANOVA). Relative changes in gene expression were analyzed by one-way ANOVA with three technical replicates per biological replicate from either greenhouse-or field-grown samples. Greenhouse biomass experiments were analyzed by a one-way ANOVA with a minimum of five biological replicates. Biomass data from the 2016 field season were analyzed by a one-way ANVOA with 16 biological replicates. Biomass data from the 2017 field season were analyzed by an ANOVA model that accounted for fixed effects of transgenic pathway, independent line, and block using eight plants/genotype for n = 5 blocks. Greenhouse photosynthetic measurements were analyzed by a one-way ANOVA, and three biological replicates per measurement and field photosynthetic measurements were analyzed by a two-way ANOVA with two plant replicates per plot and five randomized replicate blocks. All ANOVA testing was followed with a Tukey’s post-hoc test for means comparison. ANOVA tables for each analysis are included in supplementary data set 15.
