# Supplementary material for: Synthetic glycolate metabolism pathways stimulate crop growth and productivity in the field
Source: Clin Infect Dis. 2019 Jan 4;363(6422):eaat9077. doi: 10.1126/science.aat9077 (PMC7745124; doi:10.1126/science.aat9077)
Supplement: Supplementary file 1 [file SCIENCE-363-6422-aat9077-s001.pdf]

Corrected 2 August 2019; see Erratum

www.sciencemag.org/content/363/6422/eaat9077/suppl/DC1

Supplementary Materials for

Synthetic glycolate metabolism pathways stimulate crop growth and productivity in the field

Paul F. South, Amanda P. Cavanagh, Helen W. Liu, Donald R. Ort*

*Corresponding author. Email: d-ort@illinois.edu

Published 4 January 2019, *Science* **363**, eaat9077 (2019)
DOI: 10.1126/science.aat9077

This PDF file includes:

Figs. S1 to S15
Tables S1 to S3
Datasets S1 to S11 and S16 to S20

Other Supplementary Materials for this manuscript include the following:
(available at www.sciencemag.org/content/363/6422/eaat9077/suppl/DC1)

Datasets S12 to S15 (Excel)

Erratum: In the Research Article "Synthetic glycolate metabolism pathways stimulate crop growth and productivity in the field," Fig. 6 and figs. S9 and S13 and affected text have been revised to correct for an error in the statistical model that incorrectly treated individual plants rather than individual blocks of plants as replicates. In the reanalysis, the 10% increase in average seasonal biomass of plants transformed with the alternative photorespiratory pathway 3 was not statistically significant. In plants in which glycolate flux was forced through alternative pathway 3 by down-regulating transport into the native photorespiratory pathway, the average 24% increase in seasonal biomass relative to wild type was statistically significant ($P = 0.028$) sustaining the original main conclusion of this field experiment. The authors thank Ed Buckler for bringing this error to their attention.

Synthetic glycolate metabolism pathways stimulate crop growth and productivity in the field

Paul F. South[1,2], Amanda P. Cavanagh[2], Helen W. Liu[3], *Donald R. Ort[1,24,5].

Correspondence to: Donald R. Ort (d-ort@illinois.edu)

Supplementary Materials:

Fig. S1 - S15
Table S1 – S3
Dataset S1 to S20

Supplemental figures

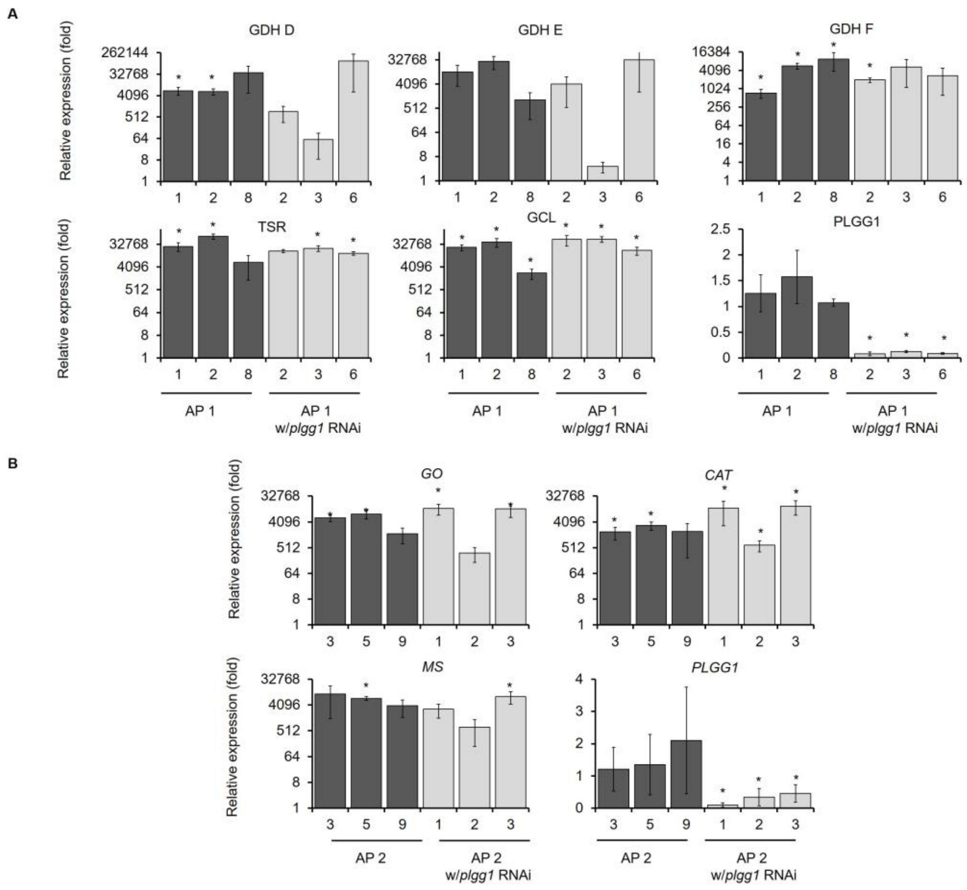

Fig. S1. Validation of transgene expression in AP1 and AP2 lines. **A.** Gene expression analysis of AP 1 and **B**. qRT-PCR analysis of the indicated transgenes and the native *PLGG1* targeted for RNAi. Error bars indicated SEM Significance based on one-way ANOVA. P≤ 0.05, values described in data set 15.

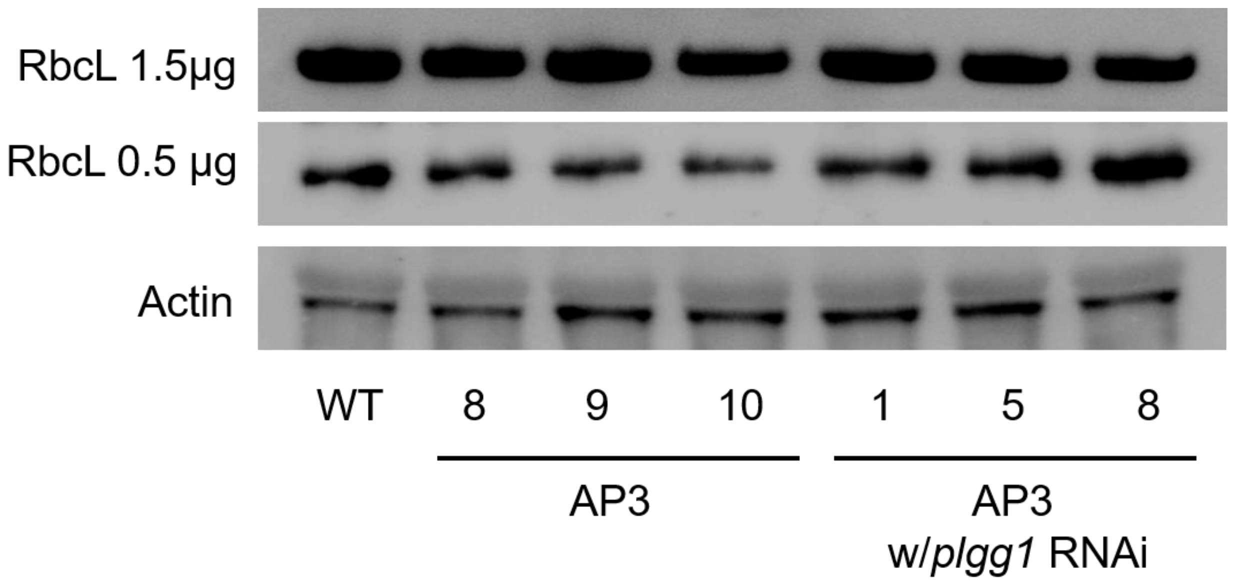

Fig. S2. Immunoblot analysis of the indicated AP3 and WT plant lines from whole leaf tissue using Actin and the large subunit of Rubisco (RbcL) antibodies. Unless indicated each lane was loaded with 5μg of total protein.

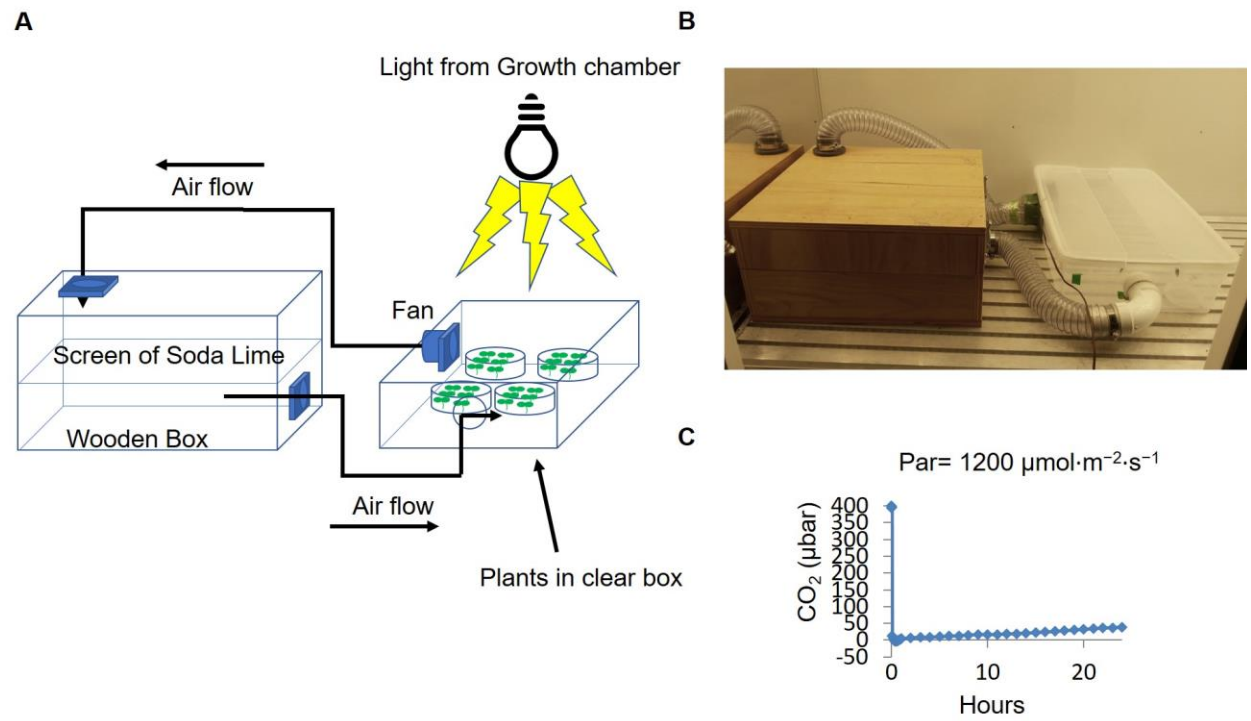

Fig. S3. Low CO_2 test chamber. **A.** Schematic of the low CO_2 test chamber. **B.** photo of test chamber within a growth cabinet. **C.** Graph represents measured CO_2 concentration during the 24 h testing period.

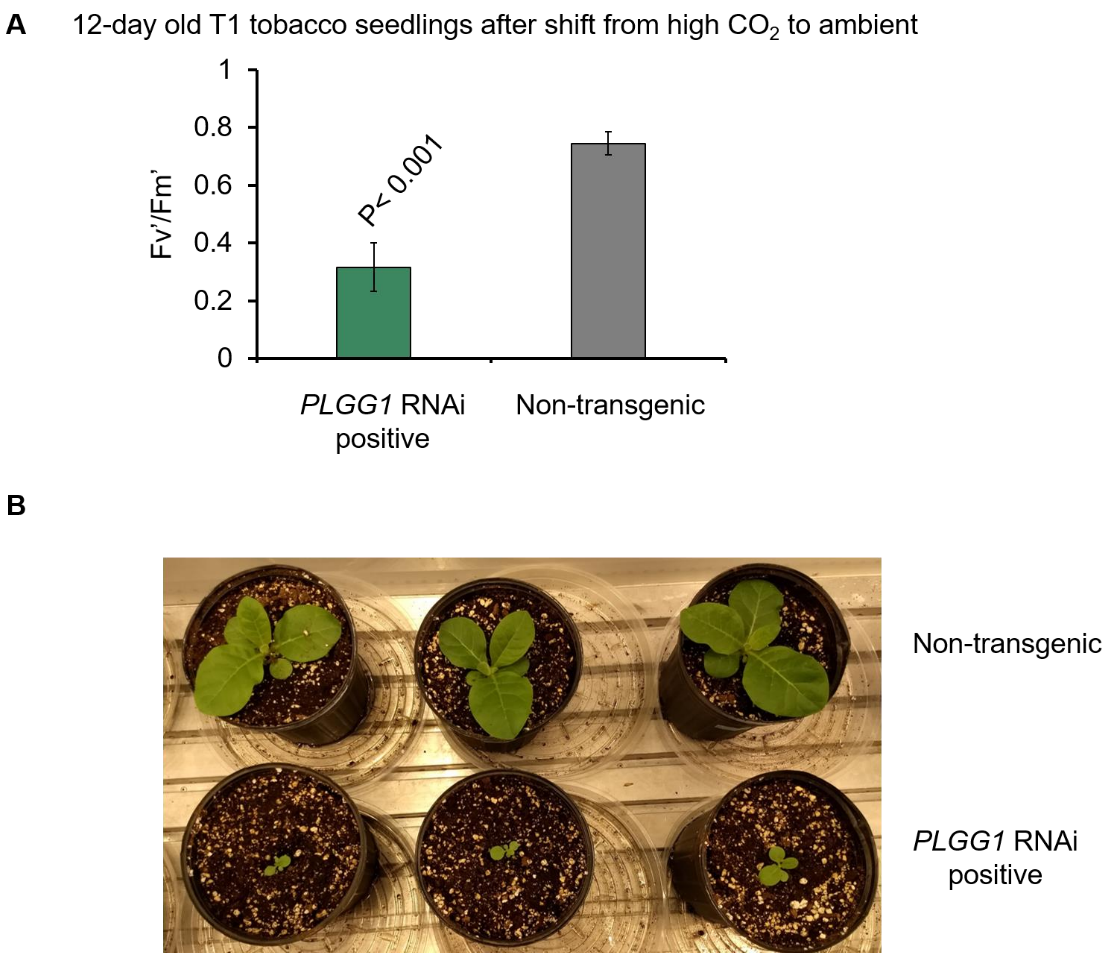

Fig. S4. RNAi targeting *PLGG1* without AP present results in a photorespiratory defect. **A.** Fv'/Fm' of transgenic positive T1 lines targeting *PLGG1* for RNAi. Plants were germinated on soil for 9 days at elevated CO_2 (2000 µbar) then transferred to ambient air for 3 days before measurement. Error bars indicate SD, P indicated in supplemental data set 15. * indicate significance based on one-way ANOVA. **B.** Photos of 3-week-old T1 plant lines with RNAi targeting *PLGG1*.

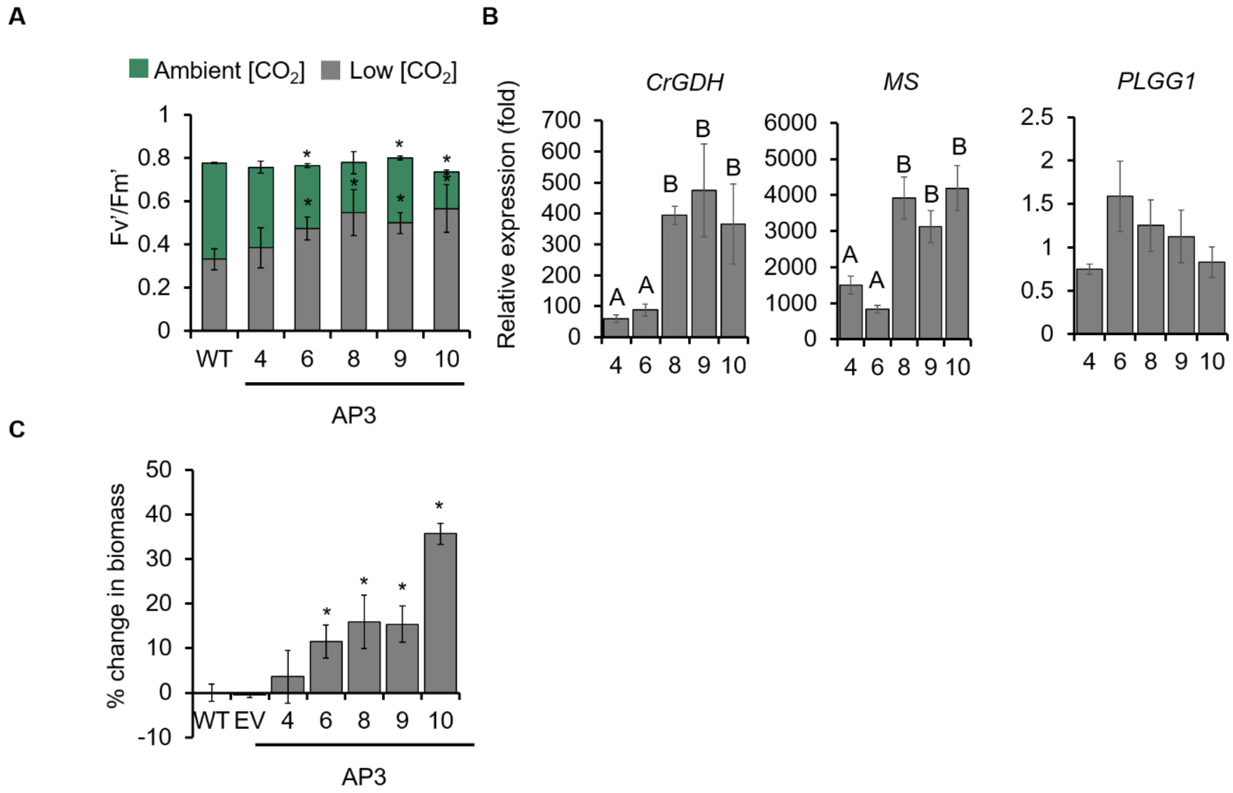

Fig.S5 Analysis of low and high expressing transgenic AP3 plant lines. **A.** Fv'/Fm' values for indicated transgenic AP3 plant line compared to WT at ambient and low [CO_2]. **B.** qRT-PCR analysis of the two transgenes in AP3 and the target gene *PLGG1* of the RNAi construct, RNA collected from the 2016 field trial. **C.** Percent difference in total dry weight biomass of the indicated combined plant lines. Error bars indicate SEM. * indicates statistical difference compared to WT based on one-way ANOVA P≤ 0.05. Fv'/Fm' for individual lines is described in supplemental data set 1 and 19. Actual significant P-values are shown in supplemental data set 15.

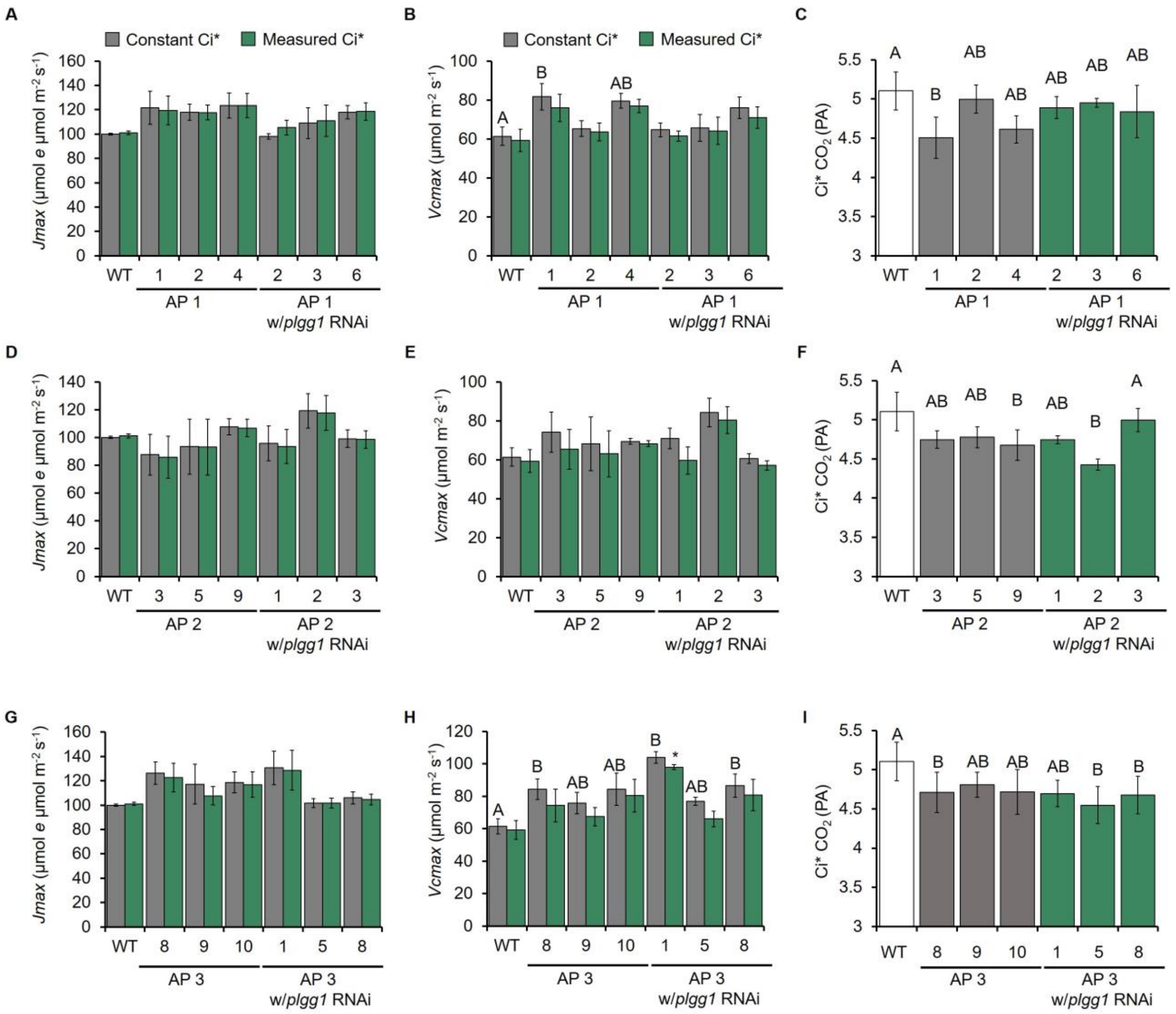

Fig. S6. Individual modelled photosynthetic parameters. **A.** J_{max}, **B.** V_{cmax}, **C.** C_i* of individual transgenic lines for AP1. **D.** J_{max}, **E.** V_{cmax}, **F.** C_i* of individual transgenic lines for AP2. **G.** J_{max}, **H.** V_{cmax}, **I.** C_i* of individual transgenic lines for AP3, data combined from H and I are used in Figure 3. For Graphs (A-B, D-E, and G-H). Apparent values of V_{cmax} and J_{max} are modelled on a constant C_i* (gray bars) and the measured C_i* values indicated in (C, F, and I) (green bars). Letters indicate statistical significance based on one-way ANOVA. P values indicated in supplemental dataset 15.

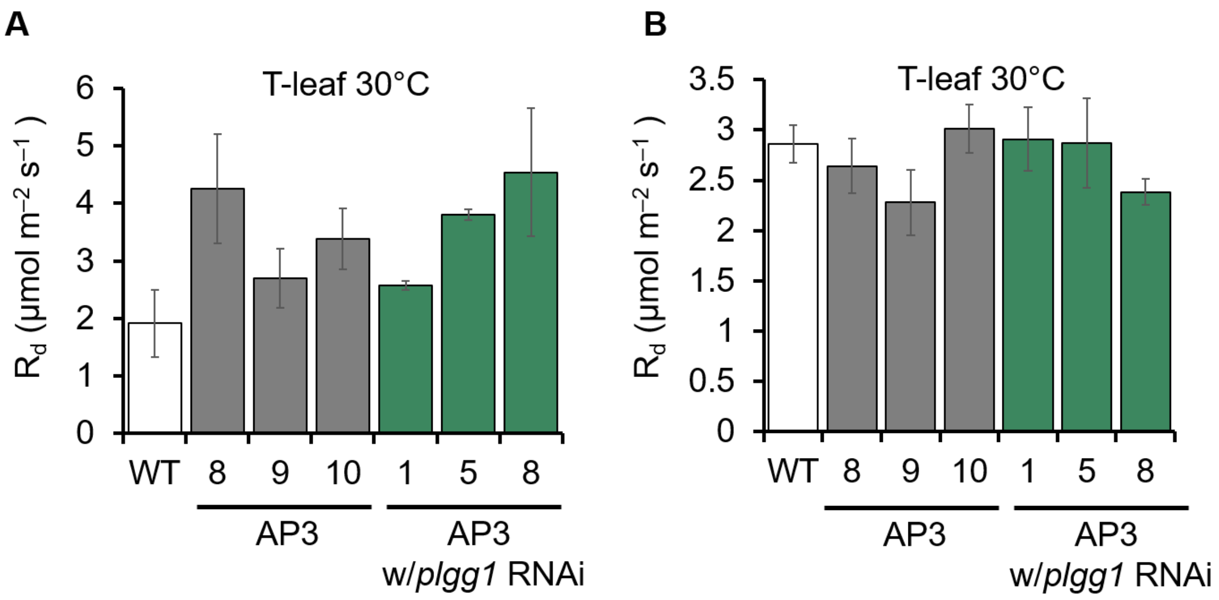

Fig. S7 Respiration rates of tested tobacco lines. **A.** Respiration measured from dark adapted leaves (Field 2017) **B.** Respiration determined from A/C_i curve analysis. Error bars indicate SEM and statistical significance is based on ANOVA analysis. P values described in dataset 15.

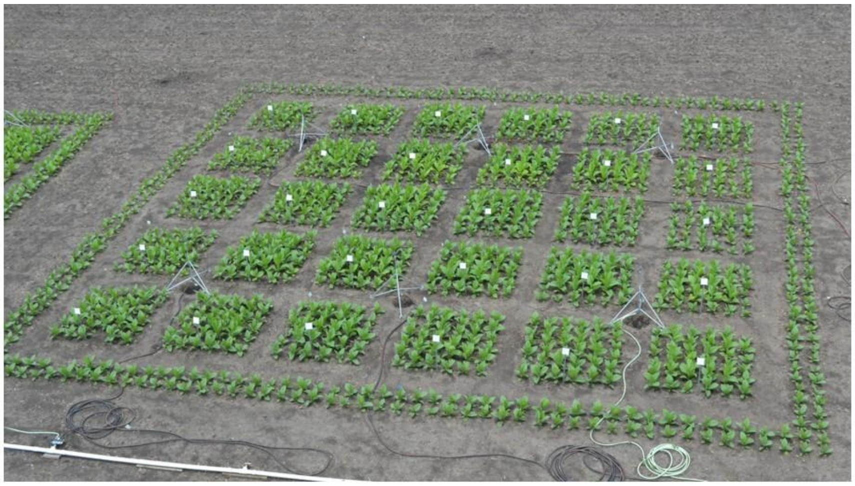

Empty Vector	AP 3 w/RNAi-5	AP 3-9	AP 1 w/RNAi-2	AP 1-2	WT
AP 3-4	AP 1-3	AP 1 w/RNAi-9	AP 3 w/RNAi-8	AP 2-3	NA
AP 3 w/RNAi-3	AP 1 w/RNAi-6	AP 1-1	AP 2 w/RNAi-2	AP 3-8	NA
AP 1 w/RNAi-3	AP 3-10	AP 3 w/RNAi-4	AP 1-4	Empty Vector -2	NA
AP 2 w/RNAi-1	AP 2-9	WT	AP 3-6	AP 3 w/RNAi-1	NA

Fig. S8. Schematic representation of 2016 field trial. The field experiment was set up as a randomized single block design. Each plant line was randomized into a single plot based on a random number generator. NA are not AP tobacco lines and were not included in this study.

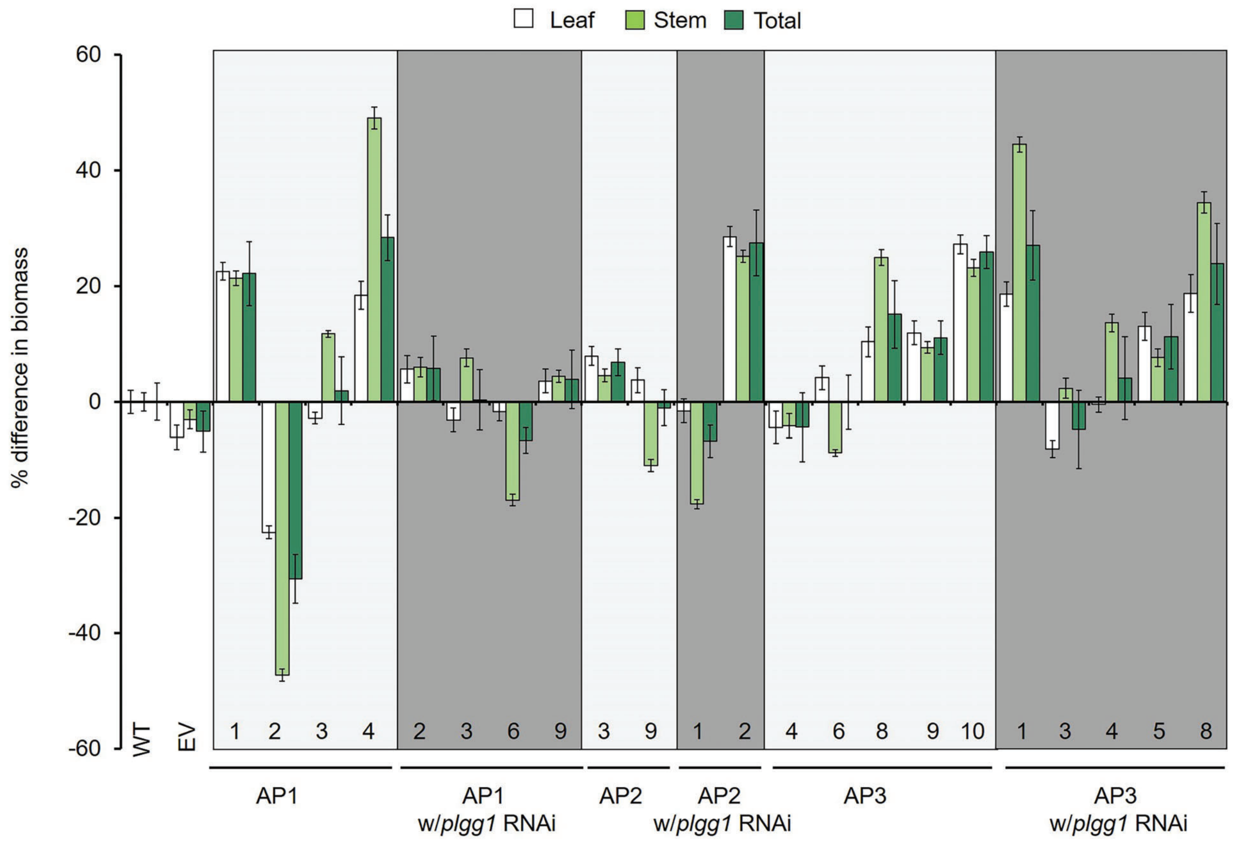

Fig. S9. Total dry weight biomass from 2016 field trials. Percent difference in combined stem, leaf, and total dry weight biomass compared to WT and empty vector (EV) controls with and without the *PLGG1* RNAi module. Error bars indicate SEM of individual lines. P values base on one-way ANOVA indicated in supplemental data set 15.

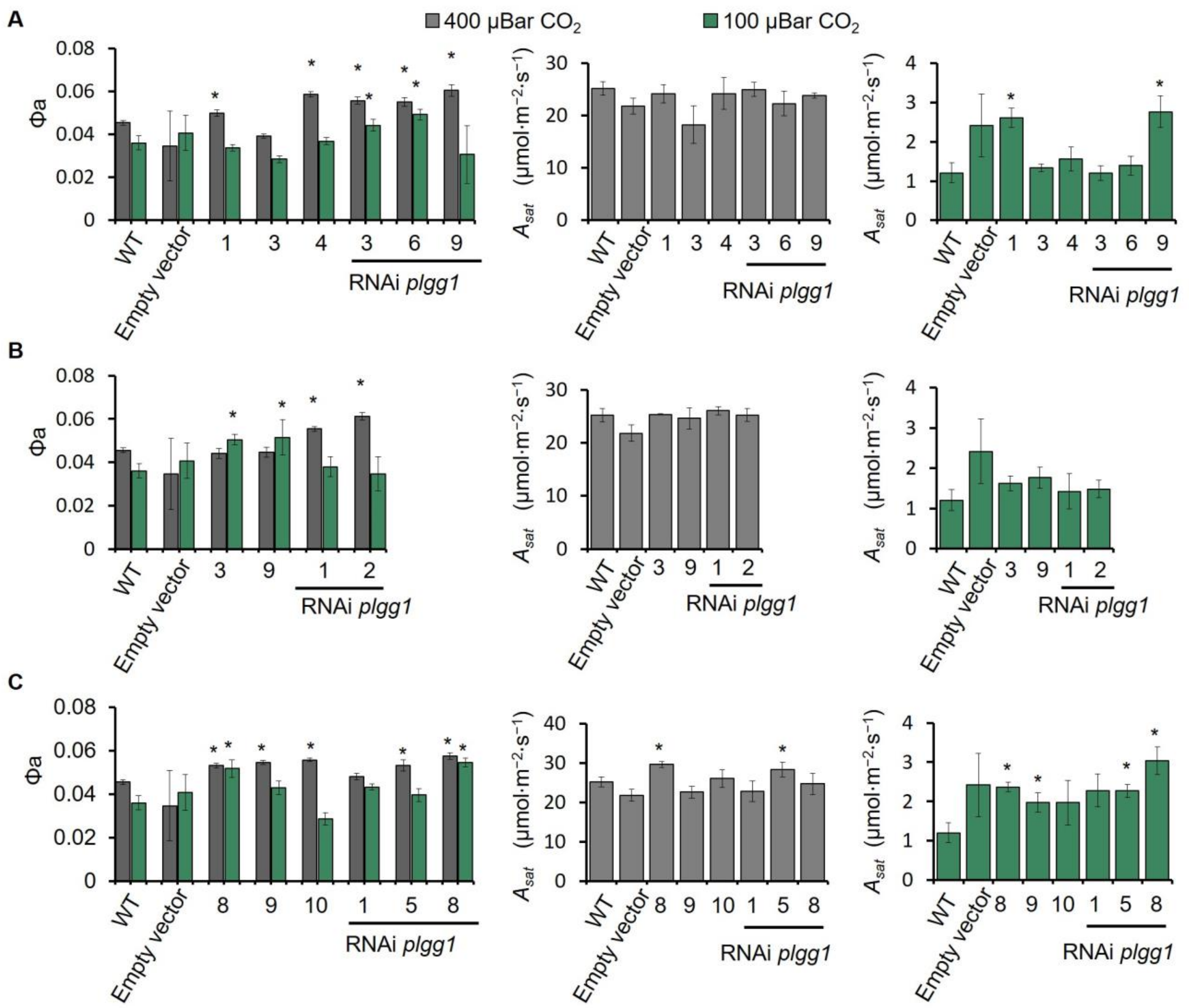

Fig. S10. Light use efficiency and saturating rates of photosynthesis from the 2016 field trials of **A.** AP1, **B**. AP2 and **C**. AP3. Combined apparent quantum efficiency of photosynthesis (Φa) determined by linear regression of assimilation based on available light response curves and saturating rates of assimilation of CO_2 at the indicated [CO_2]. Error bars indicated SEM and * indicate significance based on one-way ANOVA. P values listed in supplemental data set 15.

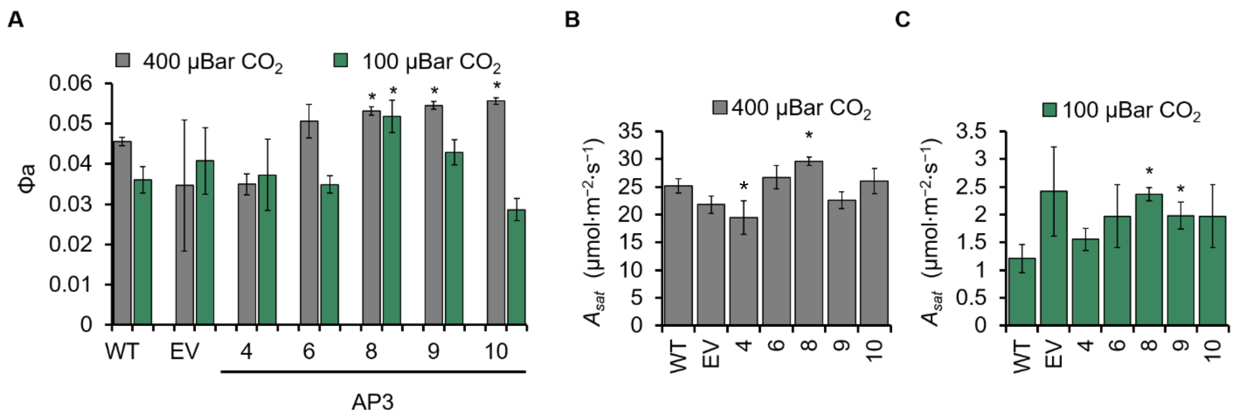

Fig. S11 Photosynthetic analysis of high and low expressing AP3 plant lines. **A.** Light use efficiency of indicated AP3 lines at 400 µBar (gray) and 100 µBar (green) [CO_2]. **B-C.** Saturating rate of photosynthesis from 2016 field trial at the indicated [CO_2]. Error bars indicated SEM and * indicate significance based on one-way ANOVA. P values listed in supplemental data set 15.

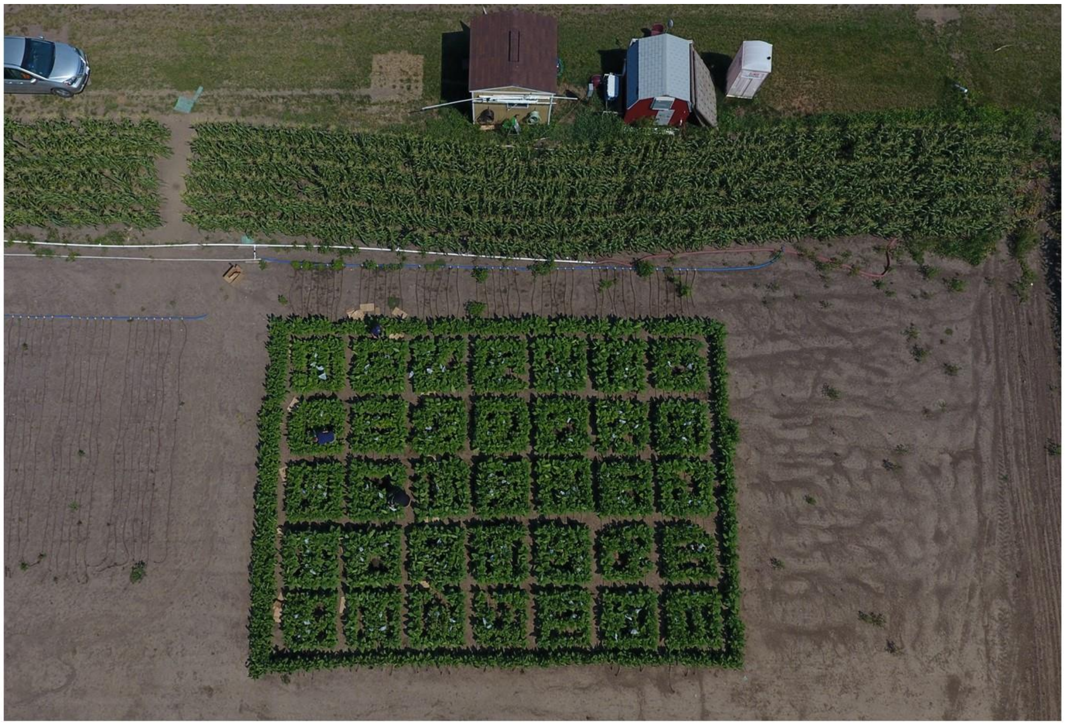

WT	AP 3 w/PLGG1 RNAi1	AP 3 w/PLGG1 RNAi-5	AP 3-8	AP 3-10	AP 3-9	AP 3 w/PLGG1 RNAi-8
AP 3-8	AP 3-10	AP 3 w/PLGG1 RNAi-8	AP 3-9	AP 3 w/PLGG1 RNAi-1	WT	AP 3 w/PLGG1 RNAi-5
AP 3 w/PLGG1 RNAi-1	AP 3-8	AP 3 w/PLGG1 RNAi-5	WT	AP 3 w/PLGG1 RNAi-8	AP 3-9	AP 3-10
AP 3 w/PLGG1 RNAi-5	AP 3-10	AP 3-9	AP 3 w/PLGG1 RNAi-8	WT	AP 3-8	AP 3 w/PLGG1 RNAi-1
AP 3 w/PLGG1 RNAi-8	AP 3-9	WT	AP 3-10	AP 3 w/PLGG1 RNAi-1	AP 3 w/PLGG1 RNAi-5	AP 3-8

Fig. S12. Schematic representation of 2017 field trial. The field experiment was set up as a randomized multiple block design. Each plant line from AP 3 was randomized into a single plot per block based on a random number generator for each block. Weather data is included in supplemental dataset 14.

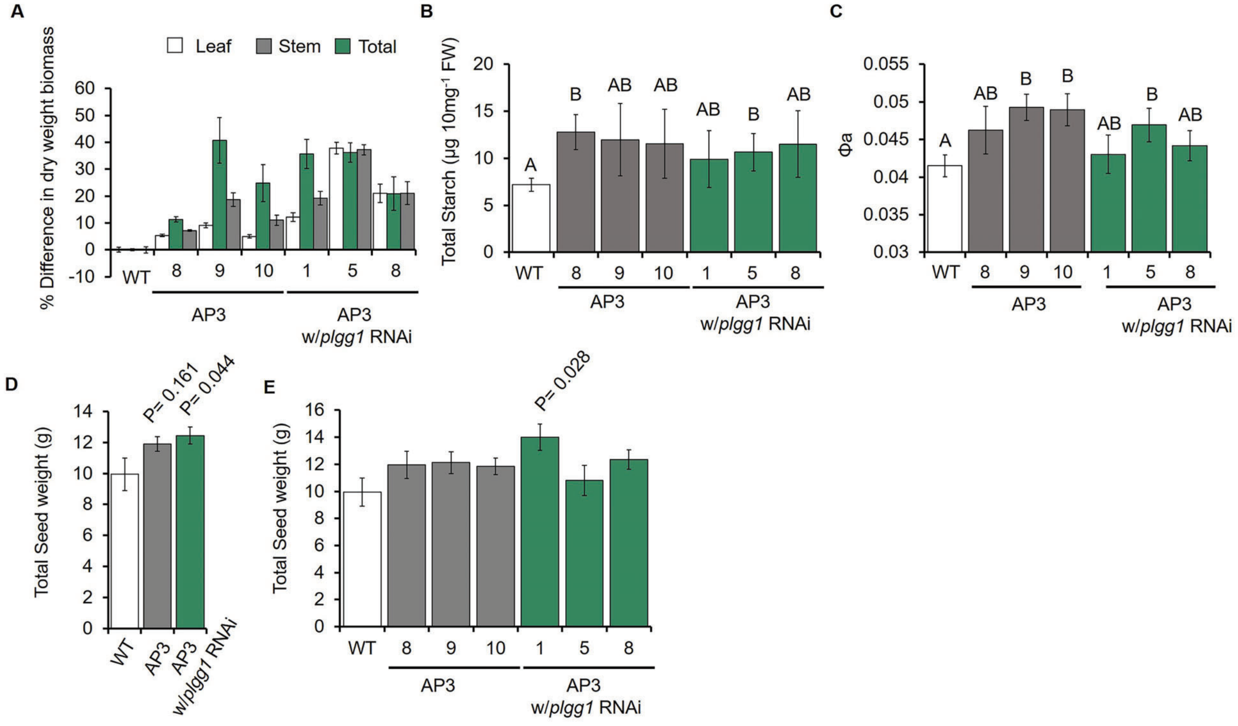

Fig. S13. Individual transgenic line data from 2017 field trial and greenhouse seed study. **A.** % difference in biomass. **B.** Total starch. **C.** Φa of individual lines for AP3. Data combined and analyzed for Fig 6. Letters indicate statistical significance based on one-way ANOVA. P≤ 0.05, values indicated in supplemental dataset 15. **D-E.** Total combined and individual seed weight from AP3 with and without RNAi greenhouse study (P values shown based on ANOVA).

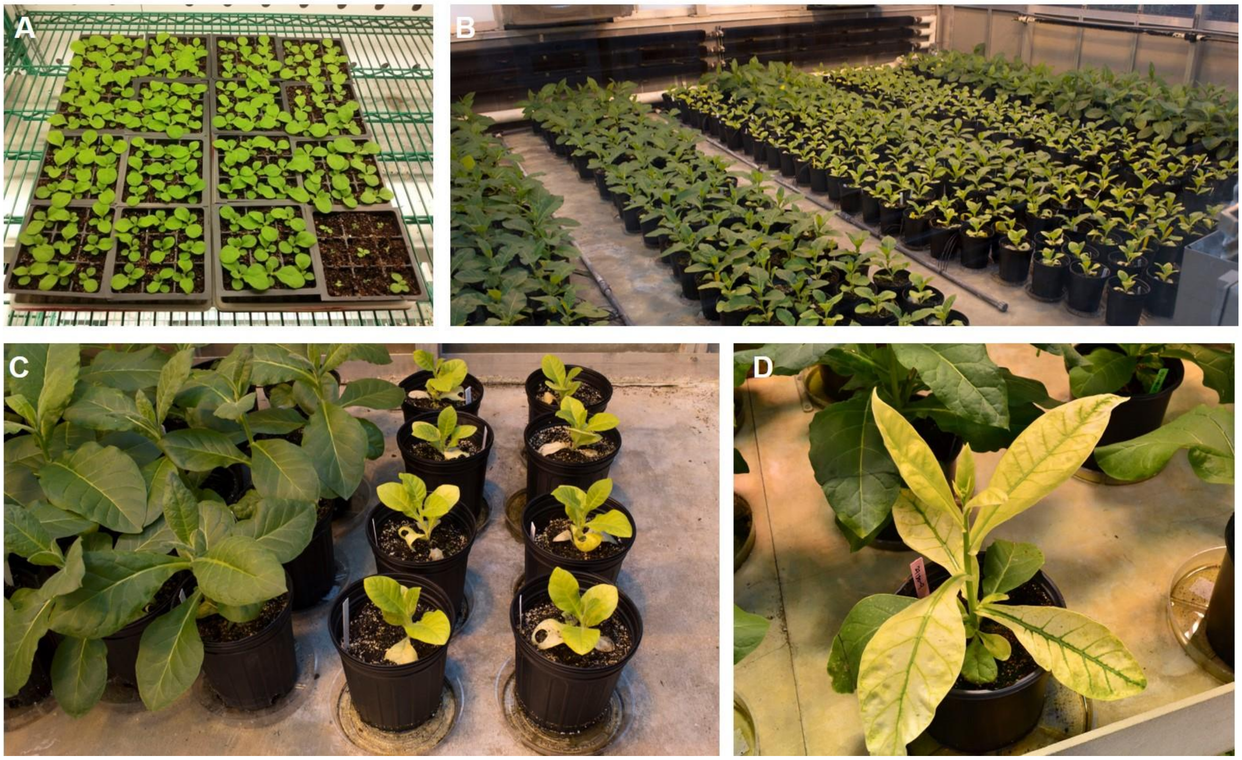

Fig. S14. Photos demonstrating that not all AP transformation events produced positive plant phenotypes. **A.** 16 independent transformation events of AP 3 with one line demonstrating defects likely based on random insertion into the genome. **B-C.** Variability in AP 2 phenotypes based on different promoter/ gene combinations. **D.** Photo representing one promoter gene combination of AP 1 that resulted in a leaf bleaching effect.

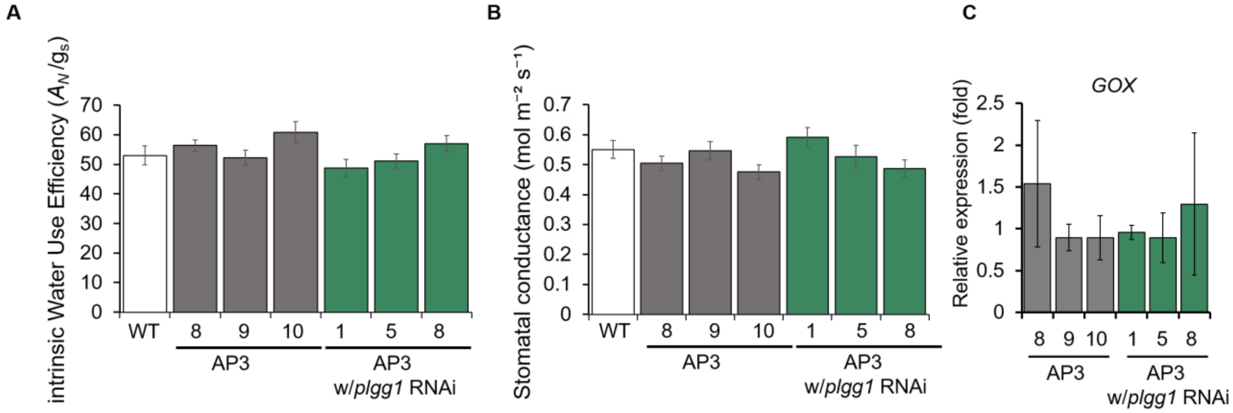

Fig. S15. Water use efficiency, stomatal conductance and glycolate oxidase expression. **A.** Determined intrinsic water use efficiency calculated from photosynthesis divided by stomatal conductance (A/g_s). **B.** Measured stomatal conductance. **C.** Relative expression of plant glycolate oxidase in the indicated plant lines. Error bares indicate SEM and significance is based on one-way ANOVA. P values indicated in dataset 15.

Supplemental Tables

Plasmid	Inserted gene	Promoter	Signal peptide	Terminator	Vector	Source
AP 1						
EC27180	TSR	Spm	RbcS	Ocs	EC50505	This Study
	GdD	RbcS	Pgm	Mas		
	GdE	Act2	RbcS	Act2		
	GdF	35s	Pgm	Act2		
	Gcl	2x35s	Pgm	35s		
EC27181	Gcl	Spm	RbcS	Ocs	EC50505	This Study
	TSR	RbcS	Pgm	Mas		
	GdD	Act2	RbcS	Act2		
	GdE	35s	Pgm	Act2		
	GdF	2x35s	Pgm	35s		
EC27182	GdF	Spm	RbcS	Ocs	EC50505	This Study
	Gcl	RbcS	Pgm	Mas		
	TSR	Act2	RbcS	Act2		
	GdD	35s	Pgm	Act2		
	GdE	2x35s	Pgm	35s		
EC27183	GdE	Spm	RbcS	Ocs	EC50505	This Study
	GdF	RbcS	Pgm	Mas		
	Gcl	Act2	RbcS	Act2		
	TSR	35s	Pgm	Act2		
	GdD	2x35s	Pgm	35s		
EC27184	GdD	Spm	RbcS	Ocs	EC50505	This Study
	GdE	RbcS	Pgm	Mas		
	GdF	Act2	RbcS	Act2		
	Gcl	35s	Pgm	Act2		
	TSR	2x35s	Pgm	35s		
EC27186	TSR	Spm	RbcS	Ocs	EC50505	This Study
	GdD	RbcS	Pgm	Mas		
	GdE	Act2	RbcS	Act2		
	GdF	35s	Pgm	Act2		
	Gcl	2x35s	Pgm	35s		
	PLGG1 RNAi	Ubi				
EC27187	Gcl	Spm	RbcS	Ocs	EC50505	This Study
	TSR	RbcS	Pgm	Mas		
	GdD	Act2	RbcS	Act2		

	GdE	35s	Pgm	Act2		
	GdF	2x35s	Pgm	35s		
	PLGG1 RNAi	Ubi				
EC27188	GdF	Spm	RbcS	Ocs	EC50505	This Study
	Gcl	RbcS	Pgm	Mas		
	TSR	Act2	RbcS	Act2		
	GdD	35s	Pgm	Act2		
	GdE	2x35s	Pgm	35s		
	PLGG1 RNAi	Ubi				
EC27189	GdE	Spm	RbcS	Ocs	EC50505	This Study
	GdF	RbcS	Pgm	Mas		
	Gcl	Act2	RbcS	Act2		
	TSR	35s	Pgm	Act2		
	GdD	2x35s	Pgm	35s		
	PLGG1 RNAi	Ubi				
EC27194	GdD	Spm	RbcS	Ocs	EC50505	This Study
	GdE	RbcS	Pgm	Mas		
	GdF	Act2	RbcS	Act2		
	Gcl	35s	Pgm	Act2		
	TSR	2x35s	Pgm	35s		
	PLGG1 RNAi	Ubi				

AP 2

Plasmid	Inserted gene	Promoter	Signal peptide	Terminator	Vector	Source
EC27171	GO	Nos	pgm	Nos	EC50505	This Study
	MS	Spm	RbcS	Ocs		
	CAT	2x35s	pgm	35s		
EC27172	CAT	Nos	pgm	Nos	EC50505	This Study
	GO	Spm	RbcS	Ocs		
	MS	2x35s	pgm	35s		
EC27173	MS	Nos	pgm	Nos	EC50505	This Study
	CAT	Spm	RbcS	Ocs		
	GO	2x35s	pgm	35s		
EC27174	GO	Nos	pgm	Nos	EC50505	This Study

	MS	Spm	RbcS	Ocs		
	CAT	2x35s	pgm	35s		
	PLGG1 RNAi	Act2		Act2		
EC27175	CAT	Nos	pgm	Nos	EC50505	This Study
	GO	Spm	RbcS	Ocs		
	MS	2x35s	pgm	35s		
	PLGG1 RNAi	Act2		Act2		
EC27176	MS	Nos	pgm	Nos	EC50505	This Study
	CAT	Spm	RbcS	Ocs		
	GO	2x35s	pgm	35s		
	PLGG1 RNAi	Act2		Act2		

AP 3

Plasmid	Inserted gene	Promoter	Signal peptide	Terminator	Vector	Source
EC27200	CrGDH	Act2	RbcS	Act2	EC50505	This study
	MS	Spm	RbcS	Ocs		
EC27201	CrGDH	Act2	RbcS	Act2	EC50505	This study
	MS	Spm	RbcS	Ocs		
	PLGG1 RNAi	RbcS		Act2		

Empty vector

Plasmid	Inserted gene	Promoter	Signal peptide	Terminator	Vector	Source
EC15325					EC50505	ENSA (project .ensa.ac.uk)
All constructs include the Basta resistance gene (Bar)	Bar	Ubi/Nos	N/A	Nos	All vectors	This study

Table S1.

Plasmid DNA constructs used in this study.

Primer Name	Primer Type	Sequence
L25 RT F	QRT-PCR	‘CCCCTCACCACAGAGTCTGC’
L25 RT R	QRT-PCR	‘AAGGGTGTTGTTGTCCTCAATCTT’
PLGG1 Nt RT-1F	QRT-PCR	‘CTCAAATAAAGTTGAAATCCTTACAAAC’
PLGG1 Nt RT-2R	QRT-PCR	‘TCTTGGTAGGGATGAATTGGAC’
RT-MS-001F	QRT-PCR	‘GGGAATCTGAGTGGACATGTG’
RT-MS-002R	QRT-PCR	‘CCAGAATTGAGTGCGTTGATG’
RT-GO-001F	QRT-PCR	‘ACAGAAACGCTTTTGCAAGG’
RT-GO-002R	QRT-PCR	‘GGTGAGCCATCTTTTGCATG’
RT-CAT-001F	QRT-PCR	‘GCGAGAAAATCACCCACTTTG’
RT-CAT-002R	QRT-PCR	‘TGGCTGGAAATAACCGTGAG’
RT-TSR-001F	QRT-PCR	‘TGAATTACTGTCGCTGGGC’
RT-TSR-002R	QRT-PCR	‘GTACAACCATTTTCACCGAACAG’
RT-GCL-001F	QRT-PCR	‘ATCAATCCGTTCTACTCAGCG’
RT-GCL-002R	QRT-PCR	‘GACATACGCCGATATTCCCTG’
RT-GdD-001F	QRT-PCR	‘GGAGGTAGCATCTTGTACGAAG’
RT-GdD-002R	QRT-PCR	‘CGGTATGCAGGATCTCAAGTC’
RT-GdE-001F	QRT-PCR	‘CGAGTGTGATTACAGCCAGG’
RT-GdE-002R	QRT-PCR	‘TGACAACGAACATCCAGCG’
RT-GdF-001F	QRT-PCR	‘CTGTGTTCACTGCGGATTTTG’
RT-GdF-002R	QRT-PCR	‘CTCCTGTGTTTTAAGCGTGAC’
RT-GOX-001F	QRT-PCR	‘TGCCGTTCACTGAAAGAGATC’
RT-GOX-002R	QRT-PCR	‘GTGGAACTAATACTTTGCCTTGG’

Table S2.

Primers used for gene expression analysis in this study.

Enzyme characteristics				
Glycolate dehydrogenase				
glycolate + acceptor ↔ glyoxylate + reduced acceptor				Source
	$\boldsymbol{K_m}$ **(mM)**	$\boldsymbol{K_{cat}}$ **(min^{-1})**	$\boldsymbol{K_{cat}/K_m}$ **(min^{-1} mM^{-1})**	***(16)***
Glycolate	0.21 ± 0.06	116 ± 7	655	
Malate Synthase				
acetyl-CoA + H_2O + glyoxylate ↔ (S)-malate + CoA				
	$\boldsymbol{K_m}$ **(mM)**	$\boldsymbol{K_{cat}}$ **(min^{-1})**	$\boldsymbol{K_{cat}/K_m}$ **(min^{-1} mM^{-1})**	***http://brenda-enzymes.org/enzyme***
Glyoxylate	0.0011-2	191.4-2886		

Table S3.
Kinetic properties of indicated enzymes.
